# The coordinate actions of calcineurin and Hog1 mediate the stress response through multiple nodes of the cell cycle network

**DOI:** 10.1371/journal.pgen.1008600

**Published:** 2020-04-28

**Authors:** Cassandra M. Leech, Mackenzie J. Flynn, Heather E. Arsenault, Jianhong Ou, Haibo Liu, Lihua Julie Zhu, Jennifer A. Benanti

**Affiliations:** 1 Department of Molecular, Cell and Cancer Biology, University of Massachusetts Medical School, Worcester, Massachusetts, United States of America; 2 Program in Bioinformatics and Integrative Biology, Program in Molecular Medicine, University of Massachusetts Medical School, Worcester, Massachusetts, United States of America; University of California San Francisco, UNITED STATES

## Abstract

Upon exposure to environmental stressors, cells transiently arrest the cell cycle while they adapt and restore homeostasis. A challenge for all cells is to distinguish between stress signals and coordinate the appropriate adaptive response with cell cycle arrest. Here we investigate the role of the phosphatase calcineurin (CN) in the stress response and demonstrate that CN activates the Hog1/p38 pathway in both yeast and human cells. In yeast, the MAPK Hog1 is transiently activated in response to several well-studied osmostressors. We show that when a stressor simultaneously activates CN and Hog1, CN disrupts Hog1-stimulated negative feedback to prolong Hog1 activation and the period of cell cycle arrest. Regulation of Hog1 by CN also contributes to inactivation of multiple cell cycle-regulatory transcription factors (TFs) and the decreased expression of cell cycle-regulated genes. CN-dependent downregulation of G1/S genes is dependent upon Hog1 activation, whereas CN inactivates G2/M TFs through a combination of Hog1-dependent and -independent mechanisms. These findings demonstrate that CN and Hog1 act in a coordinated manner to inhibit multiple nodes of the cell cycle-regulatory network. Our results suggest that crosstalk between CN and stress-activated MAPKs helps cells tailor their adaptive responses to specific stressors.

## Author summary

In order to survive exposure to environmental stress, cells transiently arrest the cell division cycle while they adapt to the stress. Several kinases and phosphatases are known to control stress adaptation programs, but the extent to which these signaling pathways work together to tune the stress response is not well understood. This study investigates the role of the phosphatase calcineurin in the stress response and shows that calcineurin inhibits the cell cycle in part by stimulating the activity of the Hog1/p-38 stress-activated MAPK in both yeast and human cells. Crosstalk between stress response pathways may help cells mount specific responses to diverse stressors and to survive changes in their environment.

## Introduction

Cells must constantly monitor their environment and correctly interpret extracellular signals such that they grow and divide only in favorable conditions. When cells attempt to divide in unfavorable conditions this often results in cell death. Therefore, the signaling pathways that sense and interpret changes in the environment are critical. Cells must detect and distinguish among a wide array of environmental stressors including oxidative stress, temperature, DNA damage, and changes in pH or osmolarity. In each of these cases, cells transiently arrest the cell cycle, while also promoting stress-specific changes in post-translational modifications and gene expression that allow cells to adapt to their new environment [1]. To mount specific responses against all of these diverse insults, cells utilize multiple signaling pathways that respond to different inputs. However, the mechanisms by which different stress-response pathways work together to coordinate cell cycle arrest and adaptation to specific stressors is not well understood.

The cell cycle is driven by a transcriptional program that is orchestrated by an interconnected network of transcription factors (TFs). Cyclical transcription established by these TFs ensures that cell cycle regulators are expressed precisely at the times their functions are needed and promotes unidirectional progression through the cell cycle [2–4]. Many environmental stressors trigger checkpoints that inhibit the activities of these TFs and impair cell cycle progression. This occurs at both the G1/S restriction point, when cells decide whether or not to commit to DNA replication, and at the G2/M transition, before cells proceed into mitosis [5].

A critical mediator of the cellular stress response is the calcium/calmodulin-activated phosphatase calcineurin (CN), which is essential for cells to survive numerous environmental insults. In mammals, oxidative stress and nutrient starvation promote the release of lysosomal Ca^2+^, which activates CN, leading to increased lysosome biogenesis and autophagy [6,7]. Similarly, a number of environmental stressors, including alkaline pH, toxic ions, and cell wall stress, trigger an influx of cytoplasmic Ca^2+^ that activates CN in budding yeast [8]. The best studied downstream effector of CN in this system is the TF Crz1, which upon dephosphorylation by CN translocates to the nucleus and activates transcription of approximately 150 target genes [9]. Crz1 targets include many regulators that feedback and shut off Ca^2+^-signaling and the CN response, which promotes adaptation to the stress. In addition to activating gene expression via Crz1, CN regulates a number of processes that change the physiology of the cell to allow it to cope with stress including protein trafficking, transcription, polarized growth, and others [8,10].

A few cell cycle regulators have also been identified as CN targets, suggesting that CN may help coordinate the stress response with cell cycle arrest [10,11]. Inactivation of the S-phase specific transcriptional activator Hcm1 by CN leads to decreased expression of its target genes, which include TFs that act at a later stage in the cell cycle [12]. However, it is not known if CN impairs cell cycle-regulated gene expression solely through inactivation of Hcm1, or if CN regulates the TF network through additional mechanisms.

Here, we sought to obtain a network level view of how CN impacts the cell cycle to better understand the mechanisms by which cells respond to cellular stresses. To this end, we analyzed the temporal response of the cell cycle-regulated transcriptome in response to CN activation and characterized the pathways mediating the CN response at each node of the cell cycle. Remarkably, we find that CN downregulates targets of multiple cell cycle TFs through distinct mechanisms. We show that CN blocks expression of G1/S genes by prolonging the activity of the stress-activated MAPK Hog1, an established inhibitor of G1/S TFs [13,14]. In contrast, CN inactivates G2/M TFs through both Hog1-dependent and -independent mechanisms. In this way, cells tailor their response to Ca^2+^-stress by coordinating the activation of CN and Hog1. Together, CN and Hog1 trigger widespread rewiring of the cell cycle-regulated transcriptional program as well as a transient cell cycle arrest that enables cells to rapidly respond and adapt to environmental stress.

## Results

### CN inhibits the cell cycle in response to stress

**Fig 1:**
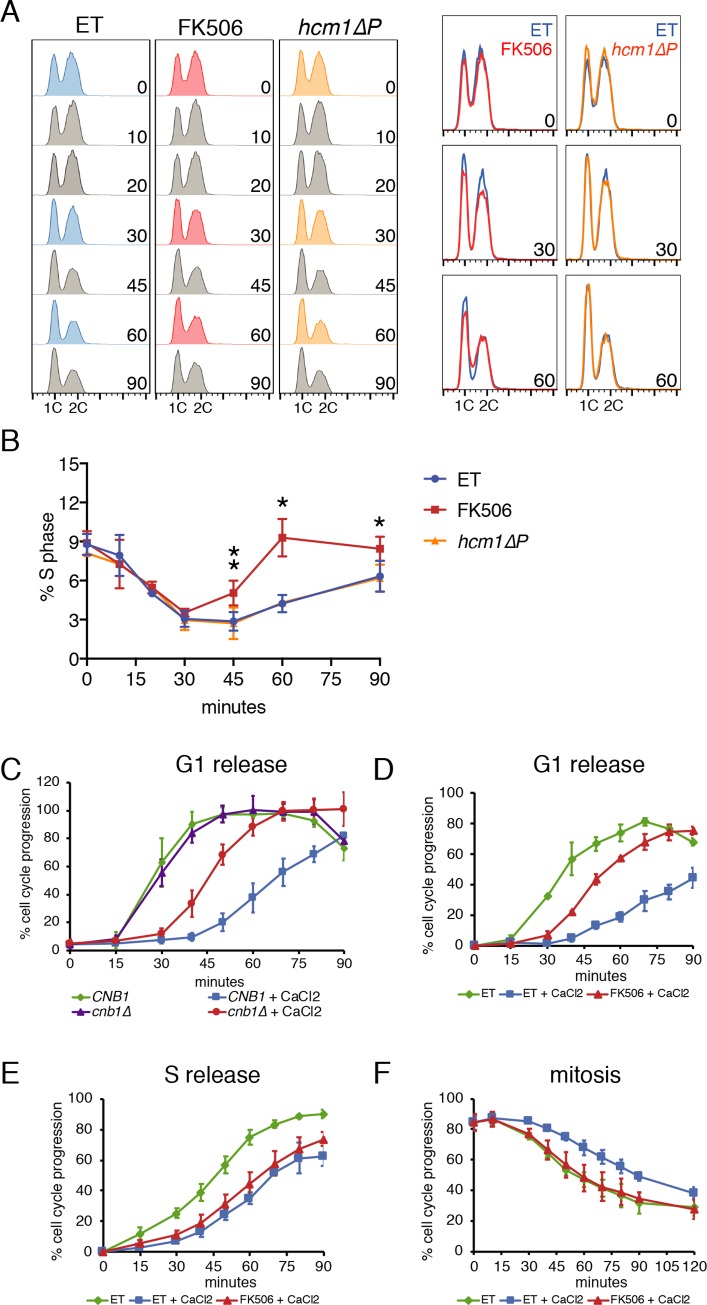
Calcineurin delays cell cycle progression. **(A)** *crz1Δ* or *crz1Δ hcm1ΔP* cells were treated with ET buffer (both gentoypes) or FK506 (*crz1Δ* only) for 15 minutes before the addition of CaCl_2_. DNA content was measured at the indicated time points using a FACScan flow cytometer. Overlays of FACS plots for selected time points are shown at the right. **(B)** Quantitation of the percentage of cells in S phase in n = 3 (ET, FK506) or n = 2 (*hcm1ΔP*) experiments is shown on the right. Error bars represent standard deviations. Statistical significance between ET- and FK506-treated *crz1Δ* samples was calculated for each time point using a paired t-test. Asterisks indicate *p<0.05, **p<0.01. **(C-F)** Cells were treated as indicated in individual panels and as outlined in S2A Fig. % cell cycle progression was calculated as described in the Materials and Methods. In each panel, an average of multiple experiments is shown and error bars represent standard deviations. Primary data for all parts of Fig 1 are included in S3 Data. (C) *crz1Δ* cells with or without *CNB1* were released from a G1 arrest. Average values are from n = 3 experiments (D) *crz1Δ* cells treated with ET buffer or FK506 were released from a G1 arrest. Average values are from n = 3 experiments. (E) *crz1Δ* cells were released from an S phase arrest. Average values represent n = 3 experiments. (F) *crz1Δ* cells were synchronized in mitosis before the addition of CaCl_2_. Average values are from n = 4 experiments.

To understand the dynamics of cell cycle regulation by CN as cells respond and adapt to stress, we followed the cell cycle distribution of a population of asynchronous cells over time following CN activation. Cells were treated with CaCl_2_ to elicit strong activation of CN, following pre-treatment with either the CN inhibitor FK506 or buffer alone to identify CN-specific changes. In addition, to lengthen the window of time before cells adapted to the CaCl_2_ stress, experiments were performed in cells lacking the CN target Crz1. In these conditions, the addition of CaCl_2_ arrested cells by 30 minutes, which was evident by the depletion of cells in S-phase (Fig 1A and 1B). Interestingly, this arrest occurred in both control (ET) and FK506-treated cells, indicating that the initiation of cell cycle arrest is CN-independent. However, at later time points, FK506-treated cells resumed cycling while control cells remained arrested (Fig 1A and 1B). A similar pattern of cell cycle arrest also occurred in wild-type (*CRZ1* proficient) cells, although cells resumed cycling more quickly, as expected (S1A and S1B Fig). These data demonstrate that the transient cell cycle arrest induced by CaCl_2_ occurs independently of CN, however CN is required to maintain the arrest over time.

It is difficult to discern whether one or multiple phases of the cell cycle are blocked in response to CN activation when experiments are performed in asynchronous cultures. To determine whether progression through one or more phases is inhibited, we assayed the consequences of CN activation in cells synchronized in specific phases (S2A Fig). First, we examined how CaCl_2_ affects cells when they are released from a G1 arrest. In the absence of CaCl_2_, cells with and without the CN regulatory subunit Cnb1 released from a G1 arrest with similar kinetics (Fig 1C, green and purple, and S2B Fig). However, when CN-expressing cells were released into the media containing CaCl_2_ there was a significant delay in progression through S phase, and this delay was partly reversed in *cnb1Δ* cells (Fig 1C, compare blue and red, and S2B Fig). Similar results were obtained when CN was inhibited by FK506 (Fig 1D and S2C Fig), and in a wild type (*CRZ1*) background (S1C and S1D Fig). This CN-dependent G1/S delay could be the result of cells arresting at the G1/S transition, or if cells proceed through the G1/S transition but CN inhibits DNA replication. To distinguish between these possibilities, cells were released from an early S-phase block (after the G1/S transition but prior to DNA replication; S2A Fig). Interestingly, although the addition of CaCl_2_ caused a modest delay in progression through S phase, there was no significant difference between control and FK506-treated cells (Fig 1E and S2D Fig). Thus, CN activation acts on the G1/S transition to delay entry into S phase.

We next tested whether CN delays progression through mitosis. Previous studies have implicated CN in arresting cells at G2/M phase through its ability to activate the Cdk inhibitor Swe1 [11,15,16]. However, these experiments were performed in cells lacking a regulator of the PP2A phosphatase (Zds1), which results in elevated levels of Swe1 and a G2/M delay even in the absence of CN activation. Importantly, we observed a CN-dependent delay when CaCl_2_ was added to Zds1-expressing cells that were synchronized in mitosis (Fig 1F and S2E Fig). Together, these results support the conclusion that CN delays cell cycle progression at multiple stages while cells adapt to environmental stress.

### CN downregulates multiple clusters of cell cycle genes

**Fig 2:**
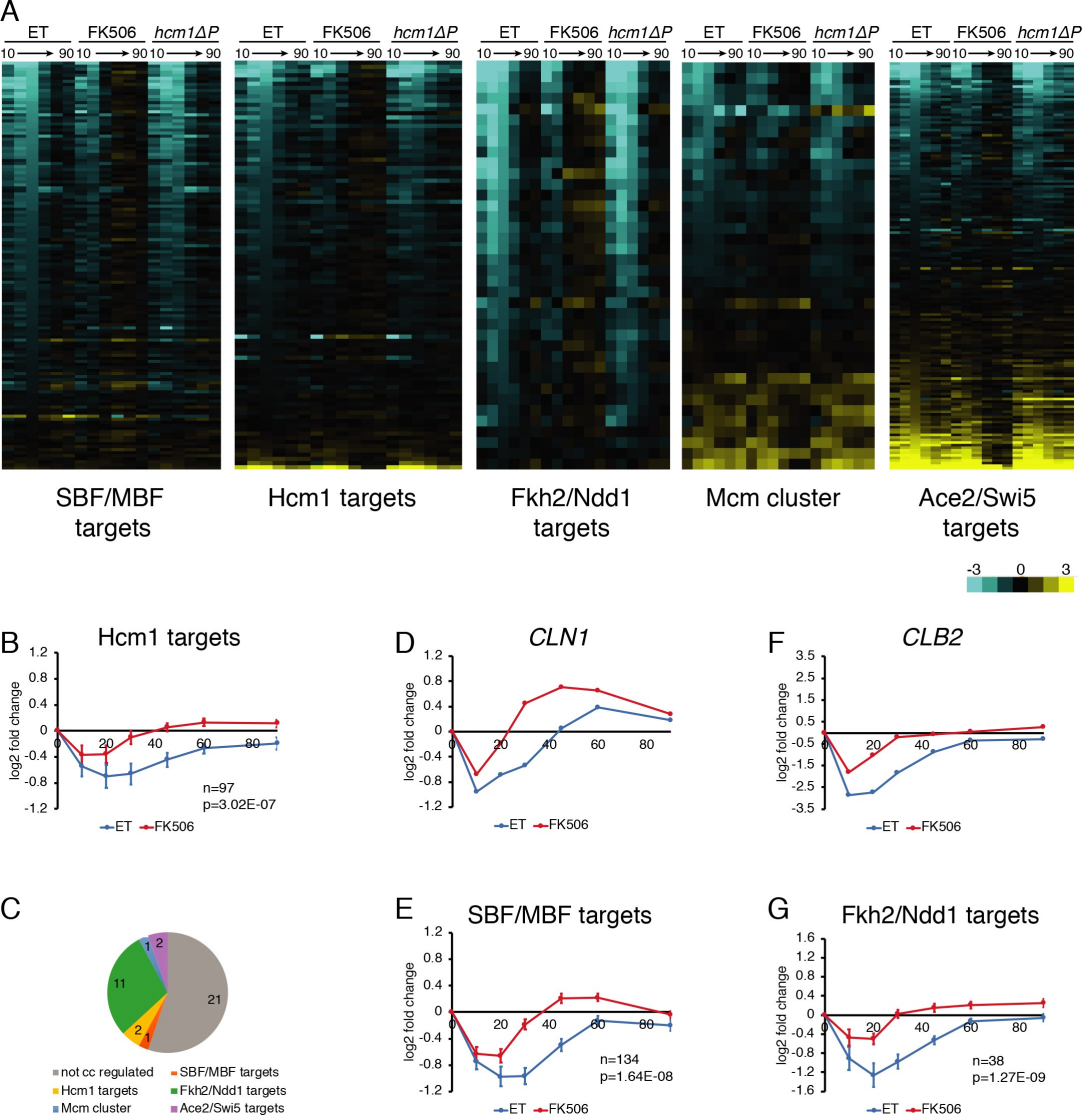
Calcineurin downregulates expression of cell cycle genes. RNA-seq was performed on duplicate time course experiments in which *crz1Δ* or *crz1Δ hcm1ΔP* cells were treated with ET buffer (both gentoypes) or FK506 (*crz1Δ* only) for 15 minutes before the addition of CaCl_2_, as in Fig 1A. **(A)** Heat maps showing log2 fold change in expression of cell cycle-regulated genes compared to each corresponding 0-minute time point. Lists of genes in each cluster and values used to generate heat maps are included in S1 Data. **(B)** Average expression of all Hcm1 target genes. Error bars indicate the 95% confidence interval. Number of genes (n) and the adjusted p-value indicating the significance of the difference between ET and FK506 curves are included. **(C)** Genes whose expression are significantly different between ET and FK506-treated *crz1Δ* cells after 10 minutes of CaCl_2_ treatment. 17 of 38 genes (colored segments) are cell cycle regulated genes. See S1 Table for list of genes. **(D)** Expression of the SBF/MBF target *CLN1* from RNA-seq data. **(E)** Average expression of all SBF/MBF target genes. Error bars indicate the 95% confidence interval. Number of genes (n) and the adjusted p-value indicating the significance of the difference between ET and FK506 curves are included. See S3 Fig for subsets of SBF/MBF targets. **(F)** Expression of the Fkh2/Ndd1 target *CLB2* from RNA-seq data. **(G)** Average expression of all Fkh2/Ndd1 target genes. Error bars indicate the 95% confidence interval. Number of genes (n) and the adjusted p-value indicating the significance of the difference between ET and FK506 curves are included.

One mechanism by which CN could promote cell cycle arrest is by downregulating expression of genes that drive the cell cycle forward. The S-phase TF Hcm1 is an established target of CN [12]; however, an Hcm1 mutant that cannot interact with CN had no effect on the CN-mediated cell cycle delay in response to CaCl_2_ (*hcm1ΔP*, Fig 1A and 1B). To determine if CN impacts the activities of other cell cycle-regulatory TFs, RNA-seq was performed on samples from asynchronous cells treated with CaCl_2_ and changes in expression of cell cycle genes were compared between control and FK506-treated cells. Interestingly, in addition to Hcm1 target genes, multiple clusters of cell cycle genes were coordinately downregulated by CN (Fig 2A–2C, S1 Data, S1 Table). Since CN also delays the cell cycle at multiple stages (Fig 1), these data suggest that CN may inhibit the cell cycle in part by regulating gene expression.

One group of genes that showed strong CN-dependent downregulation is targets of the SBF/MBF complexes [17], which peak at the G1/S transition (Fig 2A, S1 Data). For example, the G1 cyclin Cln1 was rapidly downregulated in control cells and to a lesser extent in FK506-treated cells (Fig 2D). A similar expression pattern was observed when average expression of all SBF/MBF targets was compared between ET- and FK506-treated cells (Fig 2E, S3A–S3C Fig). Genes whose expression peaks at the G2/M transition and are regulated by the Fkh2/Ndd1 complex (also called the Clb2 cluster; [18]) were also downregulated by CN (Fig 2A). Expression of the mitotic cyclin Clb2 was strongly decreased in control, but not FK506-treated cells (Fig 2F), and a similar pattern was observed among all Fkh2/Ndd1 targets on average (Fig 2G). Similar downregulation of G1/S and G2/M genes was observed when we re-analyzed a published dataset from CaCl_2_-treated wild-type cells [9] (S1E–S1G Fig), validating our findings. Importantly, CN-dependent downregulation of these gene clusters was not an indirect consequence of a difference in cell cycle position, since the largest change in expression of these genes was at the 30-minute time point (Fig 2E and 2G), when ET- and FK506-treated cells had similar cell cycle distributions (Fig 1A). Thus, CN-dependent changes in cell cycle gene expression precede CN-dependent effects on cell cycle progression.

In contrast to the coordinated downregulation of genes activated by SBF/MBF, Hcm1, and Fkh2/Ndd1, other clusters of cell cycle genes did not display a coordinate change in expression in response to stress. In particular, the Mcm [18] and Ace2/Swi5 clusters [19–21] included genes that were downregulated as well as genes that were induced in response to CaCl_2_ (Fig 2A). In the Mcm cluster, genes such as *MCM7* were downregulated (S3D Fig), similar to other cell cycle genes. However, *YGP1* and other genes were strongly induced (S3E Fig). As a result, average expression of all genes in the Mcm cluster was largely unchanged (S3F Fig). Similar observations held true for Ace2/Swi5 targets: genes such as *DSE1* were downregulated (S3G Fig), whereas *PIL1* was upregulated (S3H Fig), and average expression of all genes in the cluster was not notably changed (S3I–S3L Fig). These results suggest that the TFs that control expression of these genes may not be regulated by CN during the stress response, or that a subset of genes in these clusters are also regulated by an additional stress-responsive TF.

### CN stimulates prolonged activation of the stress-activated MAPK Hog1/p38

We next sought to understand how CN downregulates the expression of G1/S genes, since this is a key point in the cell cycle where stress signals are integrated. In budding yeast, the stress-activated MAPK Hog1 has two roles in controlling entry into S phase. Hog1 both inhibits the SBF and MBF TF complexes that drive expression of G1/S genes [13,14], and increases levels of the Cdk1 inhibitors Sic1 and Cip1, which inhibit G1 cyclin/Cdk complexes and block entry into S phase [22,23]. Notably, many stresses that activate CN also increase osmolarity [24]. This raised the possibility that CN and Hog1 might be coordinately activated in response to CaCl_2_, and that Hog1 might trigger the downregulation of G1/S genes that we observed.

**Fig 3:**
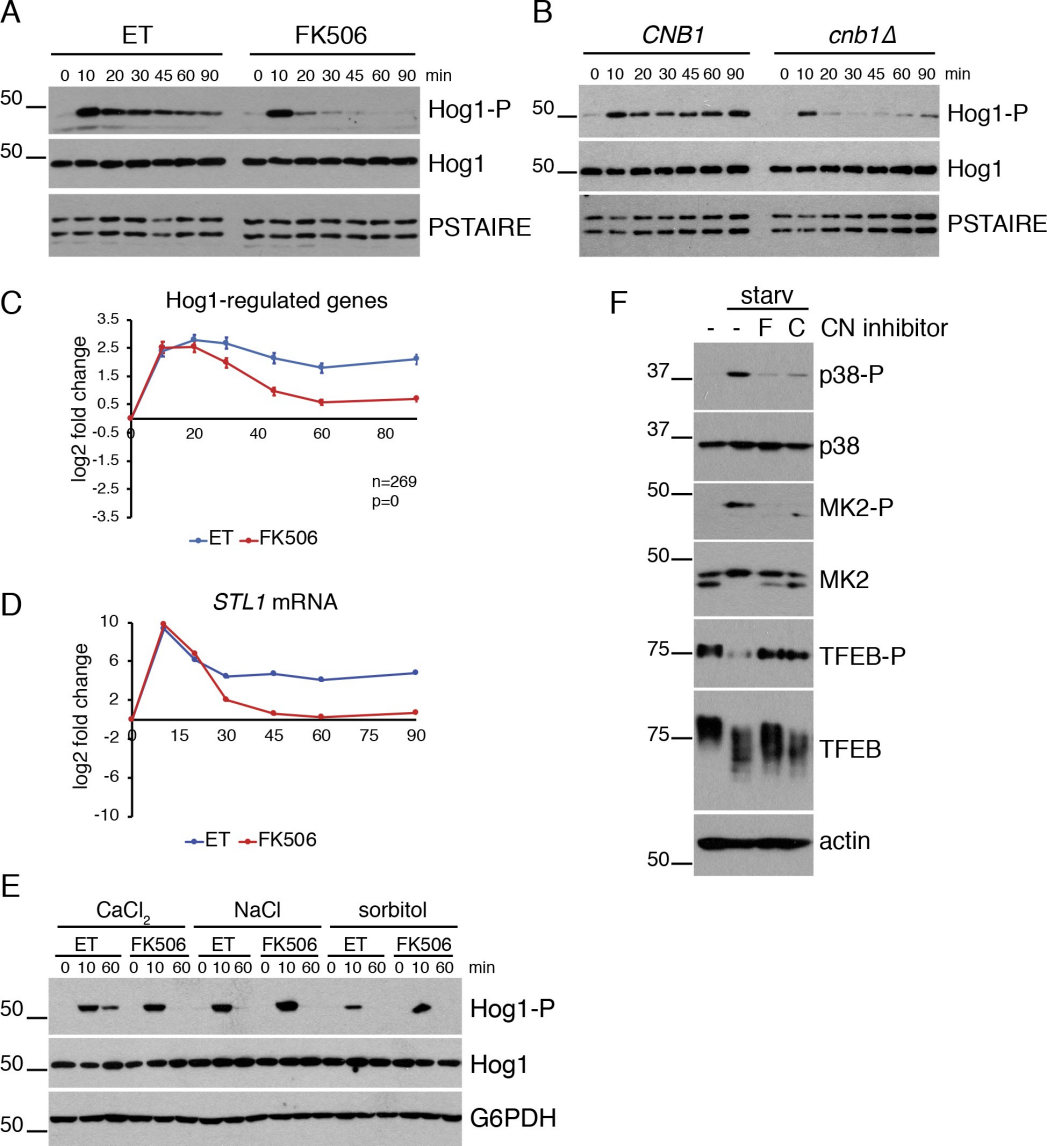
Calcineurin regulates the Hog1/p38 pathway. **(A)** *crz1Δ* cells were treated with ET buffer or FK506 for 15 minutes before the addition of CaCl_2_. The activating phosphorylation on Hog1 (Hog1-P) was monitored by Western blot. Total Hog1 levels and PSTAIRE (loading control) are shown. **(B)** *crz1Δ CNB1* and *crz1Δ cnb1Δ* cells were treated with CaCl_2_ and Hog1 activation was monitored by Western blot, as in (A). **(C)** Average expression of Hog1-regulated genes from RNA-seq experiments described in Fig 2. Error bars indicate the 95% confidence interval. Number of genes (n) and the adjusted p-value indicating the significance of the difference between ET and FK506 curves are included. **(D)** RNA was collected from cells treated as in (A) and levels of *STL1* mRNA were quantified by RT-qPCR. *STL1* levels were normalized to *ACT1* and fold change calculated relative to the 0-minute time point. Log2 fold change from a representative experiment is shown. Error bars represent the standard deviation of technical replicates. Note that error bars are too small to be visible. **(E)** Comparison of Hog1-activating stressors. *crz1Δ* cells were pretreated with ET or FK506 for 15 minutes before the addition of 200 mM CaCl_2_, 0.4M NaCl or 1M sorbitol and samples were collected after the indicated number of minutes. Shown are Western blots for activated Hog1 (Hog1-P), total Hog1 and G6PDH (loading control). Also see S4 Fig. **(F)** HFF/hTert cells were pre-treated with FK506 (F) or cyclosporin A (C) and then starved by replacing media with HBSS for 2 hours. Western blots are shown for activated p38 (p38-P), total p38, phosphorylated MK2 (MK2-P), total MK2 (upper band corresponds to phosphorylated MK2), phosphorylated TFEB (TFEB-P), total TFEB and actin (loading control).

Consistent with our hypothesis, CaCl_2_ addition led to rapid Hog1 phosphorylation that persisted over the time course (Fig 3A and 3B). In addition, expression of genes that are known to be induced following Hog1 activation [25] mirrored the timing of Hog1 phosphorylation (Fig 3C). In particular, the well-characterized Hog1-responsive gene *STL1* was induced after 10 minutes of CaCl_2_ treatment in control cells and persisted throughout the time course (Fig 3D). Interestingly, this extended period of Hog1 activation in response to CaCl_2_ differed from the reported responses to other Hog1-activating stresses, which produce a transient peak of Hog1 activity that is rapidly reduced as homeostasis is restored [26]. Indeed, although Hog1 was phosphorylated to similar extents in response to CaCl_2_, NaCl, and sorbitol, Hog1 phosphorylation was quickly reversed when cells were continually exposed to NaCl or sorbitol, whereas it persisted in CaCl_2_-treated cells (Fig 3A, 3B and 3E, S4 Fig). These results suggest that, in contrast to other osmostressors, CaCl_2_ generates an additional signal that prolongs Hog1 activation.

Consistent with this possibility, the sustained period of Hog1 phosphorylation in CaCl_2_-treated cells correlated with the persistence of CN activity, as measured by the dephosphorylation and nuclear translocation of a GFP-Crz1 reporter substrate (S5A and S5B Fig) [27]. In addition, although Hog1 was initially phosphorylated in both FK506-treated cells and cells lacking the regulatory subunit of CN, Cnb1 (Fig 3A and 3B), the CaCl_2_-induced Hog1 phosphorylation quickly decreased in the absence of CN activity, indicating that CN is required to maintain Hog1 phosphorylation in response to CaCl_2_ stress. Expression of Hog1-regulated genes mirrored the pattern of Hog1 phosphorylation, quickly returning to near starting levels when CN was inhibited, despite being induced to similar levels initially in control and FK506-treated cells (Fig 3C and 3D). A similar effect of CN on Hog1 activation was observed in a wild type (*CRZ1* proficient) strain background (S5C Fig). Together, these data demonstrate that CN is required to sustain Hog1 activation in response to continuous CaCl_2_ exposure.

Since CN and Hog1 are highly conserved proteins that each respond to a wide array of stress signals, we investigated whether crosstalk between these pathways may be conserved in other systems. CaCl_2_ stress is not well-studied in mammalian cells, however CN has been shown to be activated in response to amino acid starvation, leading to the dephosphorylation and activation of the TF TFEB, which subsequently activates autophagy [6,7]. This raised the question of whether CN can activate the Hog1-homolog p38 in human cells under these conditions. To test this, we assayed activation of p38 and CN pathways in immortalized primary human fibroblasts [28] following starvation, which is achieved by replacing the growth media with HBSS buffer for a short period of time. Interestingly, we found that starvation activated the p38 pathway, as indicated by an increase in p38 phosphorylation, and phosphorylation of its downstream substrate MK-2 (Fig 3F). In addition, TFEB phosphorylation decreased, confirming that CN was activated. Importantly, when CN was inhibited by pre-treating cells with either FK506 or cyclosporin A before starvation, p38 activation was blocked, confirming that CN activity stimulates the p38 pathway. These results suggest that crosstalk between CN and p38 pathways is conserved in human cells and is likely to occur in numerous additional biological contexts.

### CN controls Hog1 activation by disrupting negative feedback

**Fig 4:**
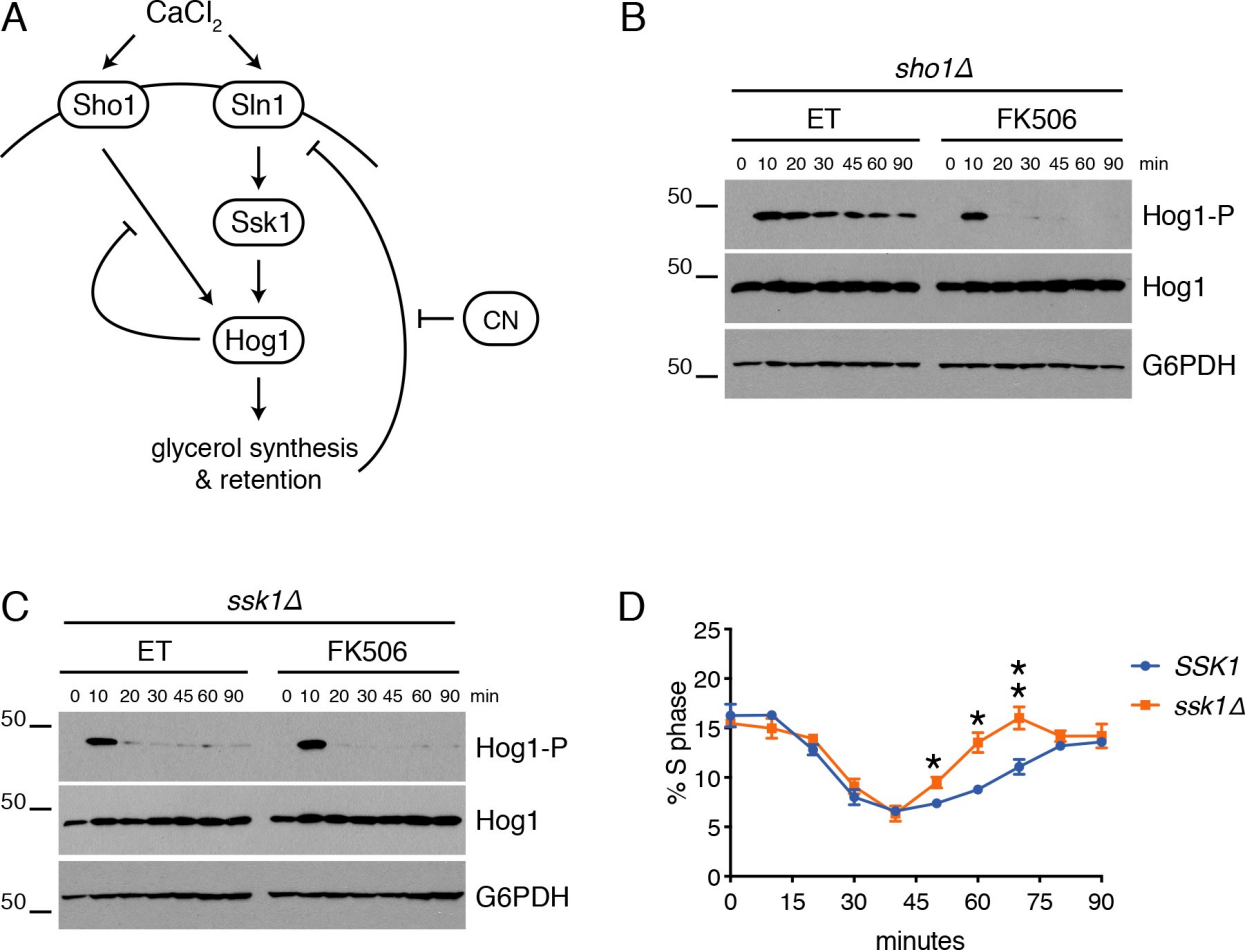
CN prolongs Hog1 activation by disrupting negative feedback. **(A)** Model of Hog1 negative feedback loops and proteins that are required for CN-Hog1 crosstalk. **(B-C)** *crz1Δ sho1Δ* (B) or *crz1Δ ssk1Δ* (C) cells were treated with ET buffer or FK506 for 15 minutes before the addition of CaCl_2_ and phosphorylated Hog1 (Hog1-P), total Hog1, and G6PDH were monitored by Western blot. **(D)** *crz1Δ* cells with (*SSK1*) or without (*ssk1Δ*) *SSK1* were treated as in (B-C) and cell cycle position monitored using a Guava easyCyte flow cytometer. Quantitation of the percentage of cells in S phase in n = 3 experiments is shown. Error bars represent standard deviations. Statistical significance between ET and FK506 samples was calculated for each time point using a paired t-test. Asterisks indicate *p<0.05, **p<0.01. Also see S6 Fig and S3 Data.

To evaluate the contribution of CN-Hog1 crosstalk to cell cycle regulation, we next sought to identify the mechanism by which CN prolongs Hog1 activation in yeast. In response to canonical osmostressors, Hog1 is activated by two upstream signaling pathways that are initiated by the Sho1 and Sln1 sensor proteins (Fig 4A). Following activation, Hog1 triggers negative feedback that shuts off both signaling cascades and limits its window of activation. First, Hog1 phosphorylates components of the Sho1 signaling pathway, including Ste50 and Sho1 itself, to inhibit their activities [29,30]. Second, Hog1 promotes an increase in intracellular glycerol, which restores turgor pressure in the cell and shuts off the Sln1 signaling cascade. This Hog1-dependent increase in glycerol is controlled by both closing of the glycerol channel Fps1 to prevent glycerol efflux [31] and promoting the activity of metabolic enzymes to stimulate glycerol synthesis [32–34]. Since Hog1 was equally activated in cells with and without CN activity, this suggested that CN might inhibit one or more of these feedback mechanisms to prolong the period of Hog1 activation in response to CaCl_2_.

To determine whether one or both of these feedback mechanisms was disrupted by CN to sustain Hog1 activation, we examined Hog1 activation in cells lacking one or the other input branch. Because *SLN1* deletion is lethal [35], the downstream regulator *SSK1* was deleted to disrupt the Sln1 signaling branch. Notably, deletion of *SSK1*, but not *SHO1*, prevented CN-dependent regulation of Hog1 phosphorylation (Fig 4B and 4C). In addition, *ssk1Δ* cells recovered from the transient CaCl_2_-induced cell cycle arrest more quickly than control cells, consistent with a more rapid dephosphorylation of Hog1 (Fig 4D, S6 Fig). These data indicate that CN prolongs Hog1 activation by delaying the restoration of turgor pressure and prolonging Sln1 signaling, which lengthens the period of stress-induced cell cycle arrest.

### Hog1 is required for CN-dependent downregulation of G1/S genes

**Fig 5:**
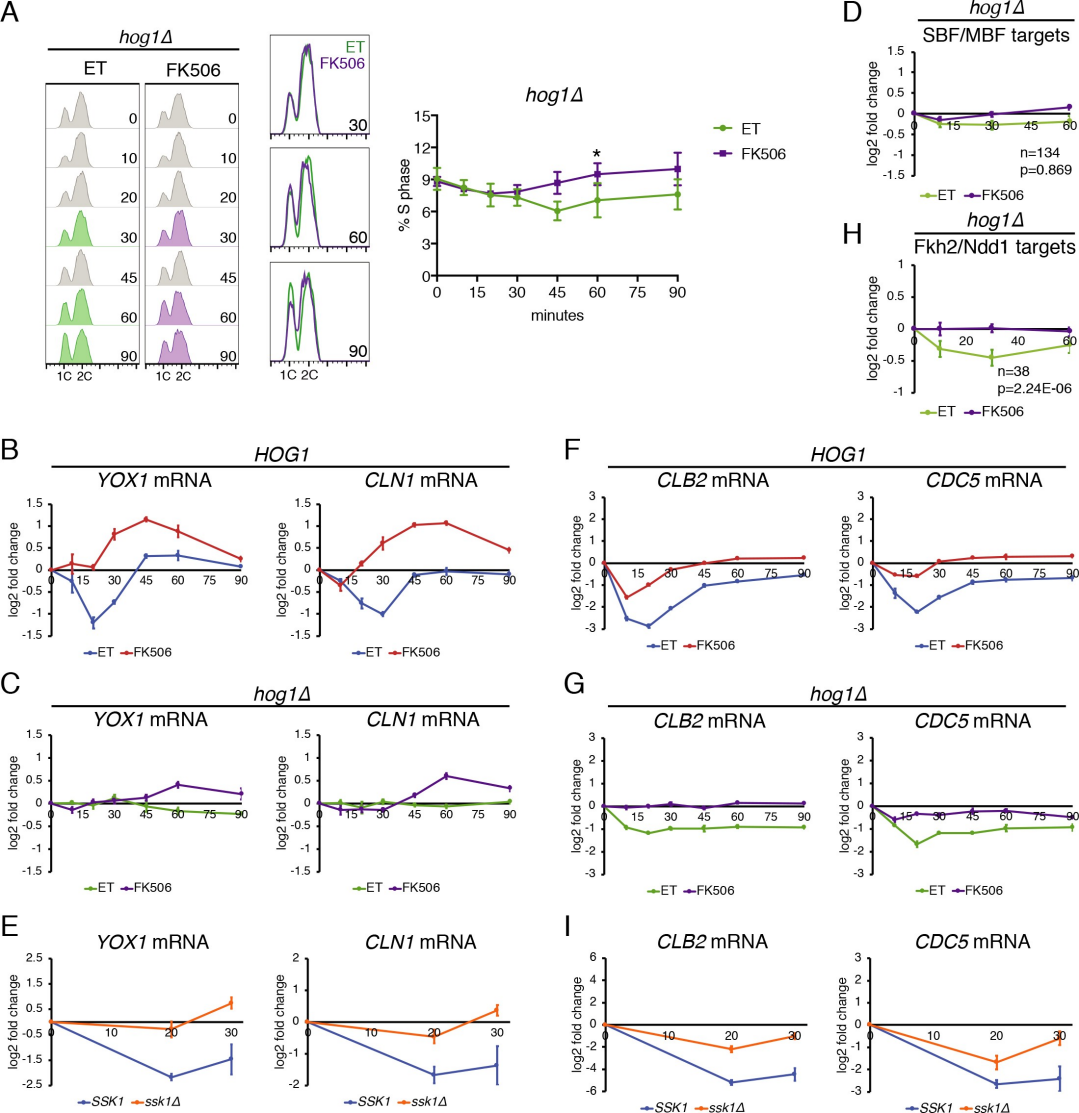
CN-dependent downregulation of G1/S genes requires Hog1. **(A)** *crz1Δ hog1Δ* cells were treated with ET buffer or FK506 for 15 minutes before the addition of CaCl_2_. DNA content was measured using a FACScan flow cytometer at the indicated time points. Quantitation of the percentage of cells in S phase in n = 3 experiments is shown on the right. Error bars represent standard deviations. Statistical significance between ET and FK506 samples were calculated for each time point using a paired t-test. Asterisk indicates *p<0.05. **(B)** RT-qPCR of SBF/MBF target genes *YOX1* and *CLN1* following the addition of CaCl_2_ to *crz1Δ* cells. Cells were pretreated with ET buffer or FK506, as indicated. Levels were normalized to *ACT1* and log2 fold change calculated relative to the 0-minute time point. Data from a representative experiment is shown. Error bars represent standard deviation of technical replicates. **(C)** RT-qPCR of *YOX1* and *CLN1*, as in (B), except experiment was performed in *crz1Δ hog1Δ* cells. **(D)** Average expression of all SBF/MBF target genes in *crz1Δ hog1Δ* cells at the indicated time points after CaCl_2_ addition, as measured by RNA-seq. Error bars indicate the 95% confidence interval. Number of genes (n) and the adjusted p-value indicating the significance of the difference between ET and FK506 curves are included. **(E)** RT-qPCR of *YOX1* and *CLN1* in *crz1Δ SSK1* and *crz1Δ ssk1Δ* cells after the addition of CaCl_2_. Average of n = 3 experiments is shown. Error bars represent standard deviations. **(F)** RT-qPCR of Fkh2/Ndd1 target genes *CLB2* and *CDC5* in *crz1Δ* cells, as in (B). **(G)** RT-qPCR of *CLB2* and *CDC5* in *crz1Δ hog1Δ* cells, as in (B). **(H)** Average expression of all Fkh2/Ndd1 target genes in *crz1Δ hog1Δ* cells, as measured by RNA-seq, as in (D). **(I)** RT-qPCR of Fkh2/Ndd1 target genes in *crz1Δ SSK1* and *crz1Δ ssk1Δ* cells, as in (E).

Having established that CN controls the duration of Hog1 activation in response to CaCl_2_, we set out to examine whether Hog1 is required for downregulation of cell cycle genes by examining the response to CaCl_2_ in *hog1Δ* cells. First, we examined the effect of *hog1Δ* on the cell cycle. Interestingly, while the fraction of *HOG1* cells in S phase decreased by 30 minutes after the addition of CaCl_2_ (Fig 1A and 1B), *hog1Δ* cells showed no significant change in cell cycle distribution after 30 minutes (Fig 5A). However, by the 45-minute time point there was a modest CN-dependent decrease in the fraction of *hog1Δ* cells in S-phase (Fig 5A). These data confirm that the initial CaCl_2_-induced arrest requires Hog1 activation, and suggest that CN maintains cell cycle arrest over time, through a mechanism that is at least partly independent of Hog1.

We next tested whether Hog1 is required for CN-dependent downregulation of SBF/MBF target genes by measuring changes in cell cycle transcripts. First, we validated the expression changes of two SBF/MBF target genes, *YOX1* and *CLN1*, which exhibited reduced expression in response to stress, as measured by RNA-seq. Both genes were downregulated within 20 minutes after the addition of CaCl_2_, and downregulation was blocked in FK506-treated cells (Fig 5B). In addition, expression of both genes was upregulated above starting levels at later time points in FK506-treated cells, similar to the average expression of all SBF/MBF target genes (Fig 2E). In contrast, CaCl_2_ did not trigger these changes in *CLN1* and *YOX1* expression in *hog1Δ* cells (Fig 5C), indicating that CN-dependent changes in G1/S gene expression require Hog1. RNA-seq analysis revealed that this pattern was consistent among the entire cluster of SBF/MBF target genes: average expression of SBF/MBF target genes did not change in *hog1Δ* cells following CaCl_2_ addition, and expression was not significantly different between control and FK506-treated cells (Fig 5D, S2 Data). Finally, we examined the regulation of G1/S genes in *ssk1Δ* cells in which CN cannot prolong Hog1 activation. Importantly, deletion of *SSK1* also eliminated CN-dependent downregulation of G1/S genes (Fig 5E). Therefore, we conclude that CN regulates SBF/MBF target genes indirectly, by controlling the duration of Hog1 activation.

We next examined whether activation of Hog1 impacted expression of Fkh2/Ndd1 target genes that peak in G2/M phase, which were also downregulated upon CaCl_2_ stress (Fig 2G). In contrast to SBF/MBF target genes, the G2/M genes *CLB2* and *CDC5*, as well as all G2/M genes in aggregate (Fig 5F–5H, S2 Data), exhibited CN-dependent downregulation in the absence of Hog1, although they were downregulated to a lesser extent than in Hog1-expressing cells. Similarly, downregulation of G2/M genes was only partially blocked in *ssk1Δ* cells in which CN-Hog1 crosstalk is disrupted (Fig 5I). These results suggest that, unlike G1/S genes, Hog1-independent mechanisms contribute to downregulation of G2/M genes.

### CN regulates levels and phosphorylation of G2/M TFs

The cell cycle regulatory TF network operates as an oscillator, with activators in each cell cycle stage inducing expression of downstream TFs in the network [36,37]. The S-phase TF Hcm1 is part of this oscillatory network and it promotes expression of the downstream TFs Fkh2 and Ndd1 that activate genes at the G2/M transition [38]. Since we found that Fkh2/Ndd1 target genes were downregulated by CN, this raised the possibility that Hcm1 inactivation by CN leads to a failure to properly induce *FKH2* and *NDD1* expression, causing a subsequent reduction in expression of Fkh2/Ndd1 target genes. However, Fkh2/Ndd1 target genes were among the earliest genes downregulated in a CN-dependent manner (Fig 2C; S1 Table). Following 10 minutes of CaCl_2_ treatment, 38 genes were significantly different between control and FK506-treated cells and 11 of these genes were Fkh2/Ndd1 targets, all of which were downregulated. This rapid decrease in expression suggests that downregulation of Fkh2/Ndd1 target genes does not depend upon decreased transcription of *FKH2* and *NDD1*, since the effects of reduced expression of the TFs on their target genes would likely require longer than 10 minutes. To test this possibility directly, levels of Fkh2/Ndd1 target genes were examined in *hcm1ΔP* cells, which express an Hcm1 mutant that lacks the CN-docking site and cannot be inactivated by CN [12]. Notably, Fkh2/Ndd1 targets were downregulated nearly identically in *hcm1ΔP* cells and ET-treated control cells (Fig 2A). This result confirms that the activity of Fkh2/Ndd1 is not indirectly inactivated via CN targeting of Hcm1 and suggests that CN regulates these TFs through an independent mechanism.

**Fig 6:**
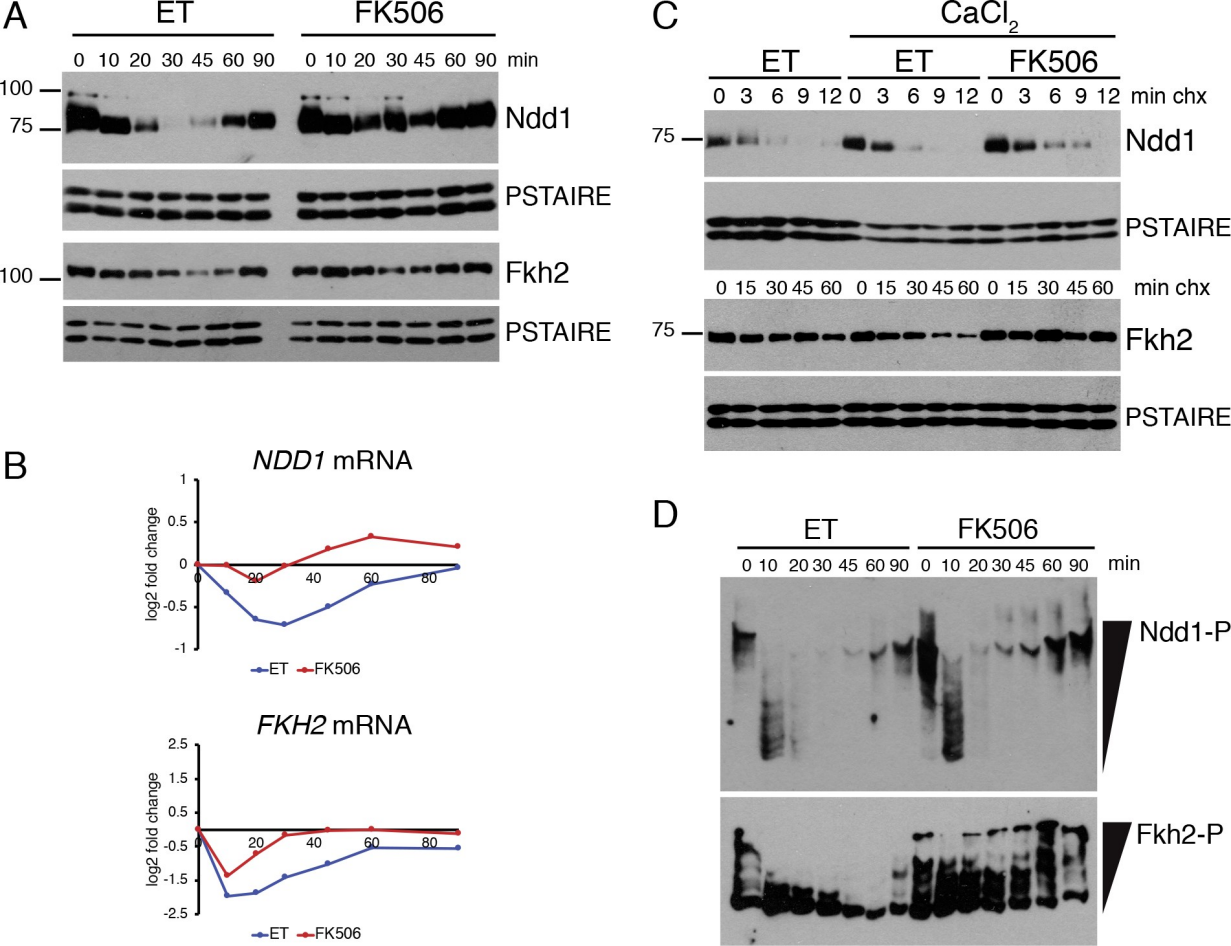
Regulation of G2/M TFs by CN. **(A)** *crz1Δ* strains were pretreated with ET buffer or FK506 for 15 minutes before the addition of CaCl_2_. Samples were collected for Western blotting at the indicated time points. Western blots were performed for a 3V5 tag on Ndd1 or a 3FLAG tag on Fkh2. PSTAIRE blots are shown as loading controls. **(B)** Expression of TF mRNAs in response to CaCl_2_. Shown are log2 fold change values, compared to the 0-minute time point, from RNA-seq experiments described in Fig 2. **(C)** Cycloheximide-chase assays of Ndd1 and Fkh2. Cells expressing 3V5-tagged Ndd1 and 3FLAG-tagged Fkh2 were pretreated with ET buffer or FK506 for 15 minutes, CaCl_2_ was added for an additional 2 minutes, then cycloheximide was added (0 minutes) and samples collected at the indicated time points for Western blot. **(D)** Phos-tag gel analysis of Fkh2 and Ndd1. ET and FK506 treated samples from experiments in (A) were run on Phos-tag gels and Western blotting performed against a 3V5 tag on Ndd1 and a 3FLAG tag on Fkh2.

To elucidate the mechanism of downregulation of Fkh2/Ndd1 target genes, we examined the levels of all known G2/M regulatory TF proteins over time following CaCl_2_ addition to control and FK506-treated cells. G2/M genes could be downregulated if expression of an activating TF is decreased, or if expression of a repressive TF is increased. Among the TFs that activate G2/M gene transcription, Ndd1 was strongly downregulated in response to CaCl_2_, and this response was largely, but not completely blocked when CN was inhibited (Fig 6A). Ndd1 protein levels mirrored its mRNA levels (Fig 6B), and Ndd1 protein exhibited a short half-life that is unchanged in response to CaCl_2_ (Fig 6C), suggesting that downregulation of Ndd1 protein results from a CN-dependent decrease in its transcription. Fkh2 protein also decreased, although to a lesser extent, after 45 minutes of CaCl_2_ treatment (Fig 6A). Although *FKH2* mRNA was strongly downregulated at the 10-minute time point (Fig 6B), the more modest change in Fkh2 protein levels is likely explained by the fact that the protein is more stable than Ndd1 (Fig 6C). We next examined the expression of the paragolous transcriptional repressors Yox1 and Yhp1, which also control expression of G2/M genes. We found that levels of Yox1 and Yhp1 decreased in response to CaCl_2_, consistent with the fact that transcription of these TFs decreases in response to CN activation (S7 Fig). Notably, although Yox1 was downregulated in both ET- and FK506-treated cells, the period of Yox1 downregulation was shortened upon CN inhibition (S7A Fig). Together these data suggest that downregulation of G2/M genes is not mediated by increased expression of repressive TFs, but results in part from decreased expression of the activating TFs Ndd1 and Fkh2.

Fkh2/Ndd1 target genes were downregulated as early as 10 minutes following the addition of CaCl_2_ (Fig 2C and 2G), however levels of Ndd1 and Fkh2 did not begin to decrease notably until later time points (Fig 6A). Therefore, other modes of regulation must also contribute to Fkh2/Ndd1 inactivation at the earliest time points after CaCl_2_ addition. Cdk1-mediated phosphorylation of both Fkh2 and Ndd1 is required for the recruitment of Ndd1 to promoters [39–41], so we examined whether either protein was dephosphorylated in response to CaCl_2_. Notably, both proteins were rapidly dephosphorylated following CaCl_2_ exposure (Fig 6D). Ndd1 was dephosphorylated in both control and FK506-treated cells, although highly phosphorylated protein began to re-accumulate by 45 minutes when CN was inhibited. In contrast, Fkh2 dephosphorylation was largely blocked when CN was inhibited by FK506 (Fig 6D), as well as in *cnb1Δ* cells (S8 Fig). Taken together, these data suggest that G2/M genes are initially downregulated as a result of dephosphorylation of Ndd1 and/or Fkh2, and this downregulation is enforced at later time points by a decrease in TF expression.

### CN regulates G2/M TFs through Hog1-dependent and -independent pathways

We next sought to disrupt the dephosphorylation and downregulation of Ndd1 and Fkh2 in response to CaCl_2_, to test whether regulation of these TFs is required for downregulation of G2/M genes. To accomplish this, we first investigated how phosphorylation of Fkh2 and Ndd1 is regulated in response to CaCl_2_. Two possibilities were considered: CN could directly dephosphorylate Fkh2 or Ndd1, or alternatively, Cdk1 activity could be inhibited and this could lead to a decrease in phosphorylation of the TFs. Ndd1 is unlikely to be a direct CN substrate, since it was dephosphorylated to a considerable degree in FK506-treated cells (Fig 6D). In contrast, Fkh2 dephosphorylation was blocked by FK506. In addition, Fkh2 contains a PAISIS motif that matches the conserved CN docking site sequence and is in an accessible region of the protein [10], making it a good candidate substrate of CN. However, we found no evidence that Fkh2 could be directly dephosphorylated by CN *in vivo* or *in vitro*.

**Fig 7:**
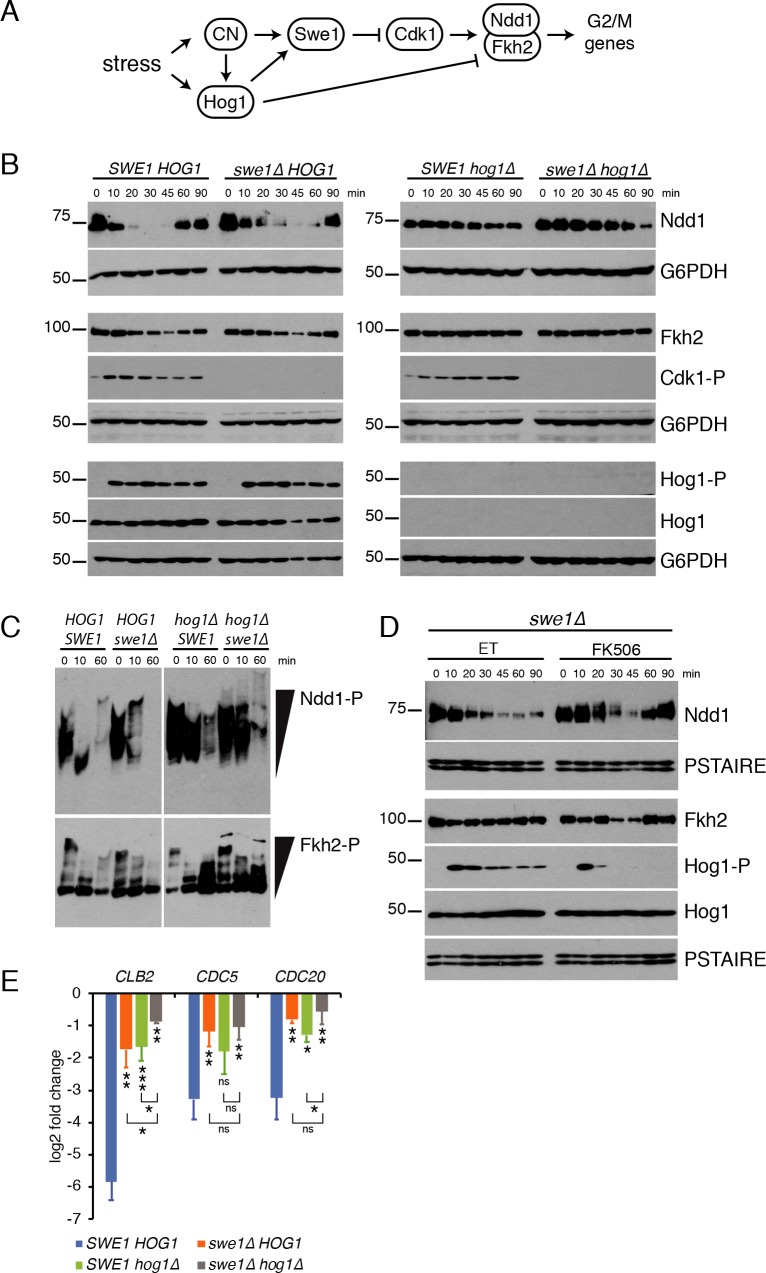
Hog1 and Swe1 regulate G2/M TF phosphorylation and activity. **(A)** Model of regulators that control G2/M TF activity in response to stress. **(B)** CaCl_2_ was added to *crz1Δ* cells with the indicated genotypes and cells were collected at the indicated time points for Western blot. Samples were assayed with antibodies against 3V5-tagged Ndd1, 3FLAG-tagged Fkh2, Cdk1 phosphorylated on Y19 (Cdk1-P), phosphorylated Hog1 (Hog1-P), total Hog1, and G6PDH (loading control). **(C)** Phos-tag gel comparing phosphorylation of 3V5-tagged Ndd1 and 3FLAG-tagged Fkh2 in cells of the indicated genotypes after the addition of CaCl_2_. Note that all 4 genotypes were run on the same gel to blot for each protein, however different exposures of left and right halves are presented. **(D)** *crz1Δ swe1Δ* cells were pretreated with ET or FK506 for 15 minutes before the addition of CaCl_2_ and samples were collected at the indicated times for Western blot as in (B). PSTAIRE is shown as a loading control. **(E)** RT-qPCR of representative Clb2 cluster genes 20 minutes after the addition of CaCl_2_ in cells with the indicated genotypes. Data is represented as log2 fold change compared to mRNA levels before the addition of CaCl_2_. Shown is an average of n = 3 experiments, error bars represent standard deviations. Statistical significance was calculated using a paired t-test, *p<0.05, **p<0.005, ***p<0.001. Asterisks beneath each bar indicated a significant difference compared to *SWE1 HOG1*, brackets indicate significance between single and double mutants.

Since downregulation of G2/M gene expression was partly dependent upon Hog1, we examined whether dephosphorylation or downregulation of Ndd1 or Fkh2 occurred in *hog1Δ* cells. Notably, Fkh2 and Ndd1 protein levels remained constant in *hog1Δ* cells after the addition of CaCl_2_ stress (Fig 7B) and dephosphorylation of Ndd1 was partially blocked (Fig 7C). In contrast, Fkh2 dephosphorylation was unaffected. These data suggest that Hog1 contributes to the G2/M TF regulation, however Hog1-independent mechanisms are also involved.

Next, we considered whether CN and/or Hog1 might inhibit Cdk1 activity to decrease phosphorylation of G2/M TFs. This is a likely possibility, since both CN and Hog1 have been reported to inhibit Clb2/Cdk1 activity indirectly through their regulation of Hsl1 [10,11,42]. Hsl1 is an inhibitor of the kinase Swe1, which phosphorylates Cdk1 on Y19 to inhibit Cdk1 activity [43]. Therefore, inactivation of Hsl1 by either CN or Hog1 is thought to promote Swe1 activity, leading to Cdk1 inhibition in response to stress (Fig 7A). Consistent with this proposed role, we observed that levels of Y19-phosphorylated Cdk1 increased upon CaCl_2_ treatment, and this phosphorylation was completely eliminated upon deletion of *SWE1* (Fig 7B). We next tested whether the Hsl1-Swe1 pathway is required for dephosphorylation of Fkh2 and/or Ndd1 upon CN activation. Notably, Ndd1 dephosphorylation was partially inhibited in *swe1Δ* cells, and Fkh2 dephosphorylation was delayed compared to controls (compare 10 min time points, Fig 7C). However, *swe1Δ* did not prevent downregulation of TF proteins. (Fig 7C and 7D). These data suggest that dephosphorylation of G2/M TFs is regulated by both Swe1-dependent and–independent pathways. Consistent with this possibility, downregulation of G2/M genes was partly blocked in *swe1Δ* cells (Fig 7E). Altogether, these data indicate that Cdk1 inhibition by Swe1 contributes to dephosphorylation of Fkh2 and Ndd1 in response to CaCl_2_ stress.

Having established that both Hog1 and Swe1 contribute to inactivation of G2/M TFs by CN, we next deleted both genes together to determine if the combination would have a greater effect on disrupting regulation of these proteins in response to CaCl_2_. Consistent with this prediction, neither protein decreased in levels and dephosphorylation was largely (though not entirely) blocked in *hog1Δ swe1Δ* cells (Fig 7B and 7C). Importantly, deletion of either *SWE1* or *HOG1* largely prevented downregulation of the G2/M genes *CLB2*, *CDC5* and *CDC20*, and *swe1Δ hog1Δ* cells showed a slightly stronger effect on the same targets, although this difference did not reach statistical significance for all comparisons (Fig 7E). These data support the conclusion that CN promotes G2/M TF inactivation through both Hog1-dependent and -independent mechanisms.

## Discussion

Many stress response pathways target cell cycle-regulatory TFs that control key transitions. Since cell cycle-regulatory TFs are part of an oscillatory and interconnected network [36,37], we set out to examine the effect of the stress-activated phosphatase CN on the entire network, and to follow changes over time as cells respond and adapt to CaCl_2_ stress. This time-resolved analysis revealed that CN has a broad role in rewiring cell cycle-regulated transcription and arresting the cell cycle. Although previous studies have identified a few direct CN substrates that impact the cell cycle [10–12], our findings suggest that many of the transcriptional and cell cycle changes that occur downstream of CN result from crosstalk to the stress-activated MAPK Hog1.

Our results present a dynamic picture of how CN and Hog1 collaborate to influence the cell cycle when cells are exposed to CaCl_2_. Initially, CaCl_2_ causes a change in osmolarity that activates Hog1, and the influx of Ca^2+^ ions into the cell activates CN. Changes in osmolarity are known to activate Hog1 within minutes, however in most cases this activation is rapidly shut off as glycerol is synthesized, its efflux is blocked, and homeostasis is restored [44,45]. We show here that CaCl_2_ affects Hog1 differently than other osmostressors, because CN provides a signal that maintains levels of active Hog1 over time.

**Fig 8:**
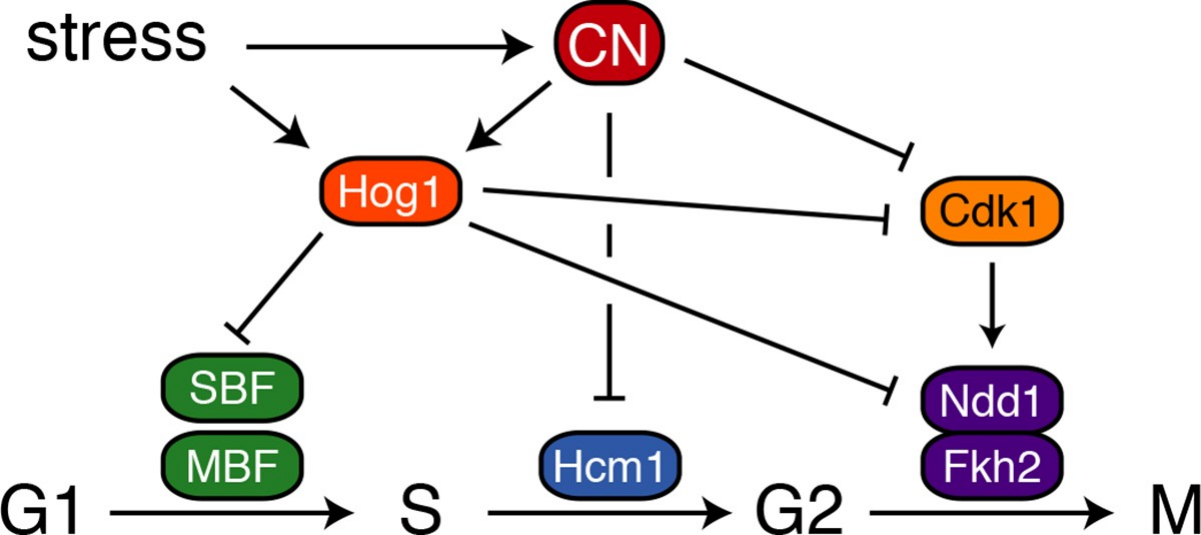
CN and Hog1 cooperate to regulate the cell cycle. Model of how CN rewires the cell cycle-regulatory TF network.

Together CN and Hog1 promote widespread changes in the expression of cell cycle-regulated genes (Fig 8). The concurrent activation of these pathways triggers an immediate downregulation of several clusters of cell cycle genes within 10 minutes of CaCl_2_ treatment: Hog1 mediates inactivation of SBF and MBF to downregulate G1/S genes [13,14], CN dephosphorylates and inactivates the S-phase TF Hcm1 [12], and CN and Hog1 independently contribute to Swe1 activation [11,42], which results in dephosphorylation of G2/M TFs Ndd1 and Fkh2. Interestingly, the specific requirements for dephosphorylation of these TFs differs: Fkh2 dephosphorylation is largely dependent on CN, whereas Ndd1 dephosphorylation may be more dependent on Hog1 and Swe1 (Figs 6D and 7C). After approximately 20 minutes, inactivation of these TFs is reinforced as CN disrupts negative feedback to maintain Hog1 activation, and levels of Fkh2 and Ndd1 proteins decrease through a Hog1-dependent pathway. Finally, after approximately 90 minutes of CaCl_2_ exposure, cells begin to adapt to the stress, levels and phosphorylation of TFs are returned to near starting levels, expression of cell cycle genes is restored, and cells resume cycling.

Since G2/M TFs Ndd1 and Fkh2 are Hcm1 target genes [38], this suggested that inactivation of Hcm1 by CN [12] might promote downregulation of G2/M genes. However, we saw no significant change in downregulation of Fkh2/Ndd1 target genes in cells expressing a CN-resistant Hcm1 protein (Fig 2A). This result is consistent with the fact that inactivating a pathway by blocking transcription is a slow response, as it depends upon shutting off expression of the TFs and waiting for the TF proteins to be degraded before having an impact on their target genes. Rapid responses are more likely to result from modulating the phosphorylation landscape, which supports our finding that the timing of Fkh2 and Ndd1 dephosphorylation correlates well with the downregulation of their target genes (Figs 5 and 6D). Downregulation of Ndd1 and Fkh2 proteins likely contributes to a decrease in target gene expression after prolonged exposure to stress.

CN not only inhibits expression of multiple clusters of cell cycle genes, but it also delays the cell cycle at multiple stages. Our findings are consistent with previous evidence suggesting that CN blocks G2/M progression through activation of Swe1. However, previous studies were carried out in a sensitized genetic background lacking the PP2A regulator Zds1 [15], and we show that this arrest also happens in Zds1-expressing cells. We also show for the first time that CN mediates a transient G1/S arrest. Our data suggests that this G1/S arrest occurs through crosstalk to the Hog1 pathway, since downregulation of SBF/MBF target genes and initial cell cycle arrest do not occur in *hog1Δ* cells. This Hog1-dependent arrest likely occurs through a combination of its effect on transcription and its ability to upregulate Cdk1 inhibitors, as previously described [13,14,22,23].

We find that CN does not impact the initial activation of Hog1, but instead prolongs the window of Hog1 activation by preventing Hog1-dependent negative feedback through the Sln1 pathway (Fig 4). The mechanism by which CN does this is not clear. One way that Hog1 impacts Sln1 signaling is by triggering the glycerol channel Fps1 to close, thereby blocking glycerol efflux [31]. Interestingly, the Fps1 regulator Ask10 was recently identified as a candidate CN target [10], suggesting it may connect CN and Hog1 pathways. However, it is also possible that CN also targets additional proteins that impact intracellular glycerol accumulation, a possibility that will be interesting to investigate in the future.

Notably, a previous study reported that Hog1 is activated by CaCl_2_, however this earlier study came to the opposite conclusion and suggested that CN promotes Hog1 dephosphorylation [46]. It is not clear why the conclusion in this previous study was different than ours, although levels of phosphorylated Hog1 were not directly compared between strains in that study, which complicates the interpretation of the data. Our results clearly show that CN activation prolongs Hog1 phosphorylation in both wild type and *crz1Δ* strain backgrounds. Moreover, we find that expression of Hog1-regulated genes directly mirrors levels of phosphorylated Hog1, and that CN activation prolongs expression of Hog1-regulated genes. These data conclusively show that CN promotes Hog1 activation.

Most environmental stresses trigger a common transcriptional response, termed the environmental stress response, which includes approximately 300 induced and 600 repressed genes [47]. However, each individual stress also activates a unique set of stress-specific changes in gene expression, which enable cells to adapt to the unique stressor. Interestingly, many environmental stressors simultaneously activate multiple signaling pathways, which may help cells tailor the specific transcriptional response to a particular stress. Hog1 and its mammalian homolog p38 in particular are activated by a wide array of stressors including heat shock, oxidative stress, glucose starvation and arsenite that also signal to other pathways [48–53]. CN is similarly activated by a number of stressors that stimulate multiple pathways. Cell wall stress activates CN in addition to the MAPK Mpk1/Slt2 [54]. Additionally, many of the canonical CN-activating stresses are ions that cause hyperosmotic stress and also activate Hog1 [24,55]. Notably, the strength and timing of CN activation differs in response to each of these stressors, which suggests that crosstalk to the Hog1 pathway may be more or less important in different conditions. In this way, crosstalk may function to tune the adaptation program and length of cell cycle arrest in response to different stressors.

Although the best understood functions of CN in mammals are in regulation of the immune system [56,57], recent evidence suggests it also plays a part in the stress response [6,7]. Oxidative stress and nutrient limitation both lead to the release of intracellular Ca^2+^ stores that activate CN. Upon activation, CN targets the transcription factor TFEB to induce expression of lysosomal biogenesis and autophagy genes. Remarkably, we find that in this context CN also stimulates p38 activation (Fig 3F). Like Hog1, p38 downregulates cell cycle-regulated gene expression and arrests the cell cycle [58], therefore CN and p38 may also work together to coordinate stress adaptation and cell cycle arrest in human cells. Crosstalk between CN and Hog1 pathways may be conserved among eukaryotes to help cells tune their response to specific types of environmental stress.

## Materials and Methods

### Yeast strains

A complete list of strains is included in S2 Table. Strains carrying gene deletions and epitope tags were constructed using standard methods [59,60]. All strains were grown in rich medium (YM-1) or synthetic complete medium (C) with 2% dextrose at 30°C [61]. In all experiments that include CaCl_2_ treatment, strains were grown in C medium with 1% ammonium chloride as the nitrogen source.

### Cell lines

Human foreskin fibroblast (HFFs) immortalized with hTERT have been previously described [28] and were maintained in Dulbecco’s modified Eagle’s medium (DMEM) containing 10% fetal bovine serum,1x Penicillin Streptomycin, 2 mM L-glutamine.

### Yeast stress experiments

Where indicated, cells were pretreated with ET buffer (90% ethanol, 10% Tween-20) or 1 μg/ml FK506 (LC Laboratories) in ET buffer 15 minutes before the addition of 200 mM CaCl_2_. For experiments with NaCl or sorbitol treatment, cells were also grown in synthetic complete medium with ammonium chloride and pretreated with ET buffer or FK506 for 15 minutes before the addition of 400 mM NaCl or 1 M sorbitol.

### Flow cytometry

Cells were fixed in 70% ethanol and stained with Sytox Green (Invitrogen) as previously described [62]. DNA content was measured on a FACScan (Becton Dickinson) or a Guava easyCyte HT (Millipore) flow cytometer. Data was analyzed using FlowJo (FlowJo, LLC) software. To quantify percent of cells in S-phase (Figs 1B, 4D and 5A; S1B, S4B and S4D Figs), percentage of cells with a DNA content between 1C and 2C was calculated. Note that the Guava easyCyte provides better resolution of the 1C and 2C peaks than the FACScan, so a higher percentage of S-phase cells in asynchronous populations is measured when samples are analyzed on the Guava. However, the fold change in percent of S-phase cells over time is similar between instruments (compare blue lines in Figs 1B and 4D). The instrument utilized for each set of experiments is noted in the corresponding figure legends. Data underlying all graphs is included in S3 Data.

### Human cell starvation experiments

Cells were seeded at 5x10^6^ cells per 15 cm dish the day before an experiment. Cells were pre-treated for 1 hour with ET buffer control (90% ethanol, 20% Tween-20), 10 μM FK506, or 5 μM cyclosporin A (LC Laboratories), washed with PBS and then starved with pre-warmed HBSS (with Ca^2+^ and Mg^+^, Gibco) with 10mM HEPES containing either ET buffer, FK506, or cyclosporin A for 2 hours.

### RNA-seq and data analysis

Total RNA was isolated from 5 OD of cells and purified as previously described [63]. Library preparation and sequencing was performed by BGI Genomic Services. Briefly, ribosomal RNAs were depleted and strand-specific libraries constructed prior to 50 base single-end sequencing on an Illumina HiSeq4000 platform. Two biological replicates of each time course were performed. All data is available in NCBI GEO and is accessible through GEO Series accession number GSE115023.

For all analyses, default parameters were used unless otherwise specified. Raw reads from RNA-seq experiments were assessed for their quality using fastqc (http://www.bioinformatics.bbsrc.ac.uk/projects/fastqc), followed by alignment to the *Saccharomyces cerevisiae* reference genome R64-1-1 from Ensembl using Tophat2 (version 2.1.1) [64] with the maximal intron length set as 100 kb. HTseq [65] was used to generate a gene-level count table, which was subsequently used for differential gene expression analysis using the voom method implemented in the limma package (version 3.32.10) [66]. Correlation within the same condition and genotype was estimated using the *duplicateCorrelation* function from the limma package.

A linear model was fit to include two independent variables, i.e., experimental condition (a combination of genotype, treatment and time post treatment) and batch, and with the correlation estimated above as input to the lmFit function. Tests of the following predefined contrasts were performed within the linear model framework. Gene expression at each time point post treatment was compared to its corresponding 0-minute baseline within the same treatment and genotype. In addition, changes of gene expression from baseline to each time point were compared between ET and FK506 treatments. Genes with a BH-adjusted p-value [67] less than 0.05 were considered as significantly differentially expressed for each comparison. Distance-based differential analyses of gene curves were performed as described [68] to compare CaCl_2_ time course data between ET and FK506-treated cells.

### Western blots

To prepare yeast samples for Western blotting, equivalent optical densities of cells were collected and lysed in cold TCA buffer (10 mM Tris pH 8.0, 10% trichloroacetic acid, 25 mM ammonium acetate, 1 mM EDTA). After incubation on ice, lysates were centrifuged and pellets resuspended in resuspension solution (100 mM Tris pH 11.0, 3% SDS). Samples were heated to 95°C for 5 minutes and clarified by centrifugation. 4X SDS-PAGE sample buffer (250 mM Tris pH 6.8, 8% SDS, 40% glycerol, 20% β-mercaptoethanol) was added to clarified lysates before heating to 95°C for an additional 5 minutes. Human cells were lysed as previously described [69] and 20-60μg of analyzed by Western blot. Western blotting was performed with antibodies against Hog1 (sc-6815 or sc-165978, Santa Cruz Biotechnology), phosphorylated Hog1/p38 (anti-Phospho-p38 MAPK, 9211L, Cell Signaling Technology), PSTAIRE (sc-53, Santa Cruz Biotechnology or P7962, Sigma), G6PDH (A9521, Sigma), V5 (R96025, Invitrogen), FLAG (clone M2, F1804, Sigma), MYC (clone 9E10, M5546, Sigma), Y19-phosphorylated Cdk1 (anti-Phospho-cdc2, 9111S, Cell Signaling Technology), p38 (9212S, Cell Signaling Technology), phosphorylated MK-2 (anti-P-MAPKAPK-2 T334 (27B7), 3007, Cell Signaling Technology), MK-2 (anti-MAPKAPK-2 (D1E11), 12155, Cell Signaling Technology), TFEB (4240S, Cell Signaling Technology), phosphorylated TFEB (anti-P-TFEB S211, 37681, Cell Signaling Technology), or actin (A1978, Sigma).

For Phos-tag gels, resolving gels contained 6% acrylamide/bis solution 29:1, 386 mM Tris-Cl, pH 8.8, 0.1% SDS, 0.2% ammonium persulfate (APS), 25 μM Phos-tag acrylamide (Wako), 50 μM manganese chloride, and 0.17% tetramethylethylenediamine (TEMED) and stacking gels contained 5% acrylamide/bis solution 37.5:1, 126 mM Tris-Cl, pH 6.8, 0.1% SDS, 0.1% APS, and 0.04% TEMED. Gels were washed twice in 100 ml of transfer buffer containing 10 mM EDTA for 15 minutes and once in 50 ml of transfer buffer for 10 minutes before transferring to nitrocellulose.

### RT-qPCR

Reverse transcription was carried out with total RNA and random primers (Promega), followed by treatment with RNase H (New England Biolabs). Quantitative PCR was preformed using 2X SYBR Fast Master Mix Universal (Kapa Biosystems) and primers for the indicated genes (see S3 Table for primer sequences) using a Mastercycler EP Realplex thermocycler (Eppendorf). mRNA levels were normalized to *ACT1* and fold change values were calculated by comparing the normalized expression at the indicated time to the expression of the target before CaCl_2_ addition.

### Cycloheximide-chase assays

Cells were treated with ET buffer or FK506 for 15 minutes before the addition of 200 mM CaCl_2_. Two minutes after CaCl_2_ addition, 50 μg/ml cycloheximide (Biomatik) was added to block protein synthesis. Samples were collected after the indicated number of minutes for Western blot analysis.

### Cell cycle synchronization

For G1 arrest-release experiments, cells were treated with 10 μg/ml alpha factor for three hours (with an additional equivalent amount re-added after 2 hours) and then released into media without alpha factor and with or without 200 mM CaCl_2_. 15 minutes prior to release, cells were treated with ET buffer or FK506 as indicated. To synchronize cells in S phase, cells were release from a three-hour alpha factor arrest into medium containing 200 mM hydroxyurea (HU) for an additional hour before releasing into medium without HU and with or without 200 mM CaCl_2_. To synchronize cells in mitosis, cells were arrested in G1 with alpha factor for 3 hours, with ET or FK506 added after 2 hours and 45 minutes. Cells were then released into media without alpha factor (containing ET/FK506) and CaCl_2_ was added 70 minutes after release from the arrest, when greater than 80% of cells had a 2C DNA content. Alpha-factor was added back 10 minutes after the addition of CaCl_2_, to block cells in the subsequent G1 phase. Percent cell cycle progression was calculated from the mean of the DNA content in each population, as previously described [70]. Data are presented as average values from a minimum of three experiments with error bars representing standard deviations.

## Supporting information

S1 Fig Regulation of the cell cycle by CN in wild type (*CRZ1*) cells. **(A)** CaCl_2_ time course in wild type (*CRZ1*-proficient) cells. Wild-type cells were treated with ET or FK506 for 15 minutes before the addition of CaCl_2_. Shown is DNA content as measured by flow cytometry at the indicated time points after CaCl_2_ addition. Colored time points are overlaid to compare ET and FK506 samples, right. **(B)** Quantitation of the percentage of cells in S phase from n = 3 experiments. Error bars represent standard deviations. Statistical significance between ET- and FK506-treated samples was calculated for each time point using a paired t-test. Asterisks indicate *p<0.05. **(C)** G1 arrest-release of wild type (*CRZ1*) cells as in Fig 1D. **(D)** % cell cycle progression was calculated for samples from (C), as described in the Materials and Methods. Average values represent n = 3 experiments, error bars indicate standard deviation. **(E-G)** Average expression of the indicated groups of cell cycle-regulated genes after the addition of 200mM CaCl_2_ to wild-type cells. Data is from [9]. Number of genes in each cluster is indicated.
(TIF)

S2 Fig Regulation of specific cell cycle phases by CN. **(A)** Schematic depicting synchronization protocols used in Fig 1C–1F and S1C and S1D Fig. **(B)** Representative FACS plots from Fig 1C. **(C)** Representative FACS plots from Fig 1D. **(D)** Representative FACS plots from Fig 1E. **(E)** Representative FACS plots from Fig 1F.
(TIF)

S3 Fig Regulation of subsets of cell cycle genes by CN. **(A-C)** Average expression of SBF/MBF target genes from Fig 2A and 2E, divided into subsets of genes regulated by both SBF and MBF (A), only SBF (B), or only MBF (C). **(D)** Expression of the Mcm cluster gene *MCM7* from RNA-seq experiments. **(E)** Expression of Mcm cluster gene *YGP1* from RNA-seq experiments. Note that *YGP1* is also a Hog1-regulated gene. **(F)** Average expression of all Mcm cluster genes shown in Fig 2A. Error bars indicate the 95% confidence interval. Number of genes (n) and the adjusted p-value indicating the significance of the difference between ET and FK506 curves are included. **(G)** Expression of Ace2/Swi5 target gene *DSE1* from RNA-seq experiments. **(H)** Expression of Ace2/Swi5 target gene *PIL1* from RNA-seq experiments. Note that *PIL1* is also a Hog1-regulated gene. **(I)** Average expression of all Ace2/Swi5 target genes shown in Fig 2A. Error bars indicate the 95% confidence interval. Number of genes (n) and the adjusted p-value indicating the significance of the difference between ET and FK506 curves are included. **(J-L)** Average expression of Ace2/Swi5 target genes from Fig 2A and (I), divided into subsets of genes regulated by both Ace2 and Swi5 (J), only Ace2 (K), or only Swi5 (L). For all parts, lists of genes and values are included in S1 Data.
(TIF)

S4 Fig CN does not prolong Hog1 activation in response to NaCl or sorbitol. *crz1Δ* cells were pre-treated with ET buffer or FK506 for 15 minutes before the addition of 0.4M NaCl **(A-B)** or 1M sorbitol **(C-D)**. Phosphorylated Hog1 (Hog1-P), total Hog1 and PSTAIRE (loading control) were monitored by Western blot (A, C) and percentage of cells in S-phase were quantified (B-D). For parts B & D, an average of n = 3 experiments are shown and error bars indicate standard deviations. Cell cycle positions were measured using a Guava EasyCyte flow cytometer.
(TIF)

S5 Fig The timing of CN and Hog1 activation in response to CaCl_2_. **(A)** *crz1Δ* cells expressing GFP fused to a portion of Crz1 that lacks the DNA binding domain (residues 14–424) were treated with CaCl_2_ for the indicated number of minutes. Dephosphorylation of the GFP-fusion protein was monitored by Western blot and confirms that CN is active throughout the 90-minute time course and correlates with the maintenance of Hog1 phosphorylation (Hog1-P). **(B)** Cells from (A) were imaged at the indicated time points to confirm that the GFP-Crz1 reporter is nuclear in most cells throughout the 90-minute time course. Cells without the GFP reporter are shown as a negative control. Scale bar represents 10μm. **(C)** Hog1 activation in wild-type cells is regulated by CN. Wild-type (*CRZ1*) cells were arrested for 3 hours in G1 with alpha factor, then pre-treated with ET buffer or FK506 for 15 minutes before releasing from the arrest into medium containing 200mM CaCl_2_. Samples were collected after the indicated number of minutes and Hog1 activation monitored by Western blot (Hog1-P). Total Hog1 and PSTAIRE (loading control) are shown.
(TIF)

S6 Fig Primary FACS data of *ssk1Δ* cells treated with CaCl_2_. FACS plots from representative CaCl_2_ time course in *crz1Δ SSK1* and *crz1Δ ssk1Δ* cells shown in Fig 4D.
(TIF)

S7 Fig Regulation of additional G2/M TFs in response to CaCl_2_. **(A)** Strains expressing the indicated tagged TFs were pretreated with ET buffer or FK506 for 15 minutes before the addition of CaCl_2_. Samples were collected for Western blotting at the indicated time points. Western blots were performed for a 3V5 tag on Fkh1, Mcm1, and Yox1 or a 13MYC tag on Yhp1. For all experiments PSTAIRE blots are shown as a loading control. **(B)** Expression of TF mRNAs in response to CaCl_2_. Shown are log2 fold change values, compared to the 0-minute time point, from RNA-seq experiments described in Fig 2. **(C)** Cycloheximide-chase assays of the indicated TF proteins. Cells expressing tagged TF proteins from (A) were pretreated with ET buffer or FK506 for 10 minutes, CaCl_2_ was added for an additional 5 minutes, then cycloheximide was added (0 minutes) and samples collected at the indicated time points for Western blot.
(TIF)

S8 Fig CN regulates dephosphorylation of Fkh2. *crz1Δ CNB1* and *crz1Δ cnb1Δ* strains were pretreated with ET buffer or FK506 for 15 minutes before the addition of CaCl_2_. Samples were collected at the indicated time points and Phos-tag Western blot performed for a 3FLAG tag on Fkh2.
(TIF)

S1 Table CN-dependent changes in gene expression after 10 minutes of CaCl_2_ stress. (PDF)

S2 Table Strain table. (PDF)

S3 Table Primer table. (PDF)

S1 Data Changes in cell cycle-regulated gene expression in response to CaCl_2_ stress. (XLSX)

S2 Data Changes in cell cycle-regulated gene expression in response to CaCl_2_ stress in *hog1Δ* cells. (XLSX)

S3 Data Quantification of FACS data. (XLSX)
