# Supplementary figures and images for: The coordinate actions of calcineurin and Hog1 mediate the stress response through multiple nodes of the cell cycle network
Source: PLoS Genet. 2020 Apr 28;16(4):e1008600. doi: 10.1371/journal.pgen.1008600 (PMC7209309; doi:10.1371/journal.pgen.1008600)

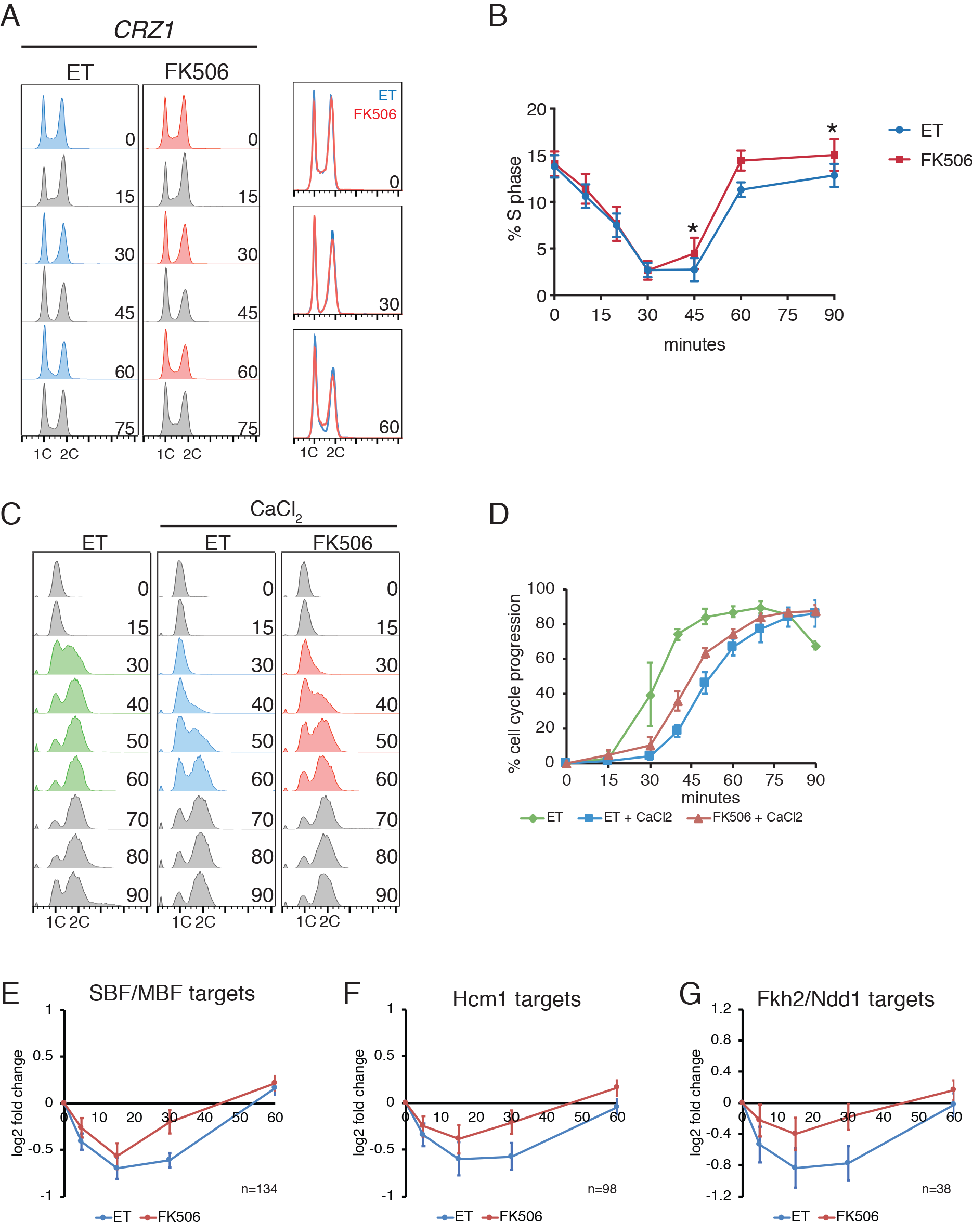

Supplement: S1 Fig — (A) CaCl2 time course in wild type (CRZ1-proficient) cells. Wild-type cells were treated with ET or FK506 for 15 minutes before the addition of CaCl2. Shown is DNA content as measured by flow cytometry at the indicated time points after CaCl2 addition. Colored time points are overlaid to compare ET and FK506 samples, right. (B) Quantitation of the percentage of cells in S phase from n = 3 experiments. Error bars represent standard deviations. Statistical significance between ET- and FK506-treated samples was calculated for each time point using a paired t-test. Asterisks indicate *p<0.05. (C) G1 arrest-release of wild type (CRZ1) cells as in Fig 1D. (D) % cell cycle progression was calculated for samples from (C), as described in the Materials and Methods. Average values represent n = 3 experiments, error bars indicate standard deviation. (E-G) Average expression of the indicated groups of cell cycle-regulated genes after the addition of 200mM CaCl2 to wild-type cells. Data is from [9]. Number of genes in each cluster is indicated. (TIF) [file pgen.1008600.s001.tif]

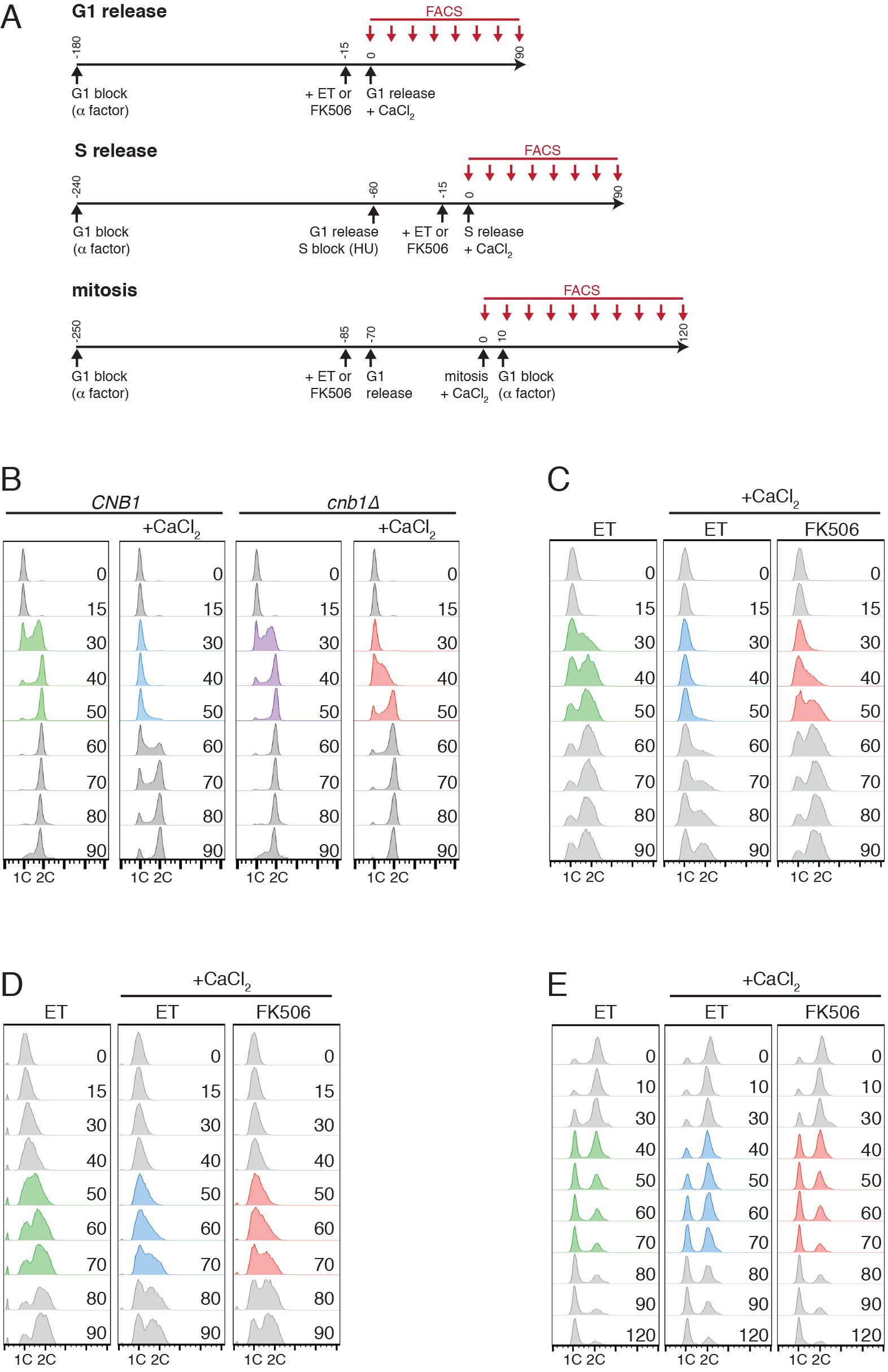

Supplement: S2 Fig — (A) Schematic depicting synchronization protocols used in Fig 1C–1F and S1C and S1D Fig. (B) Representative FACS plots from Fig 1C. (C) Representative FACS plots from Fig 1D. (D) Representative FACS plots from Fig 1E. (E) Representative FACS plots from Fig 1F. (TIF) [file pgen.1008600.s002.tif]

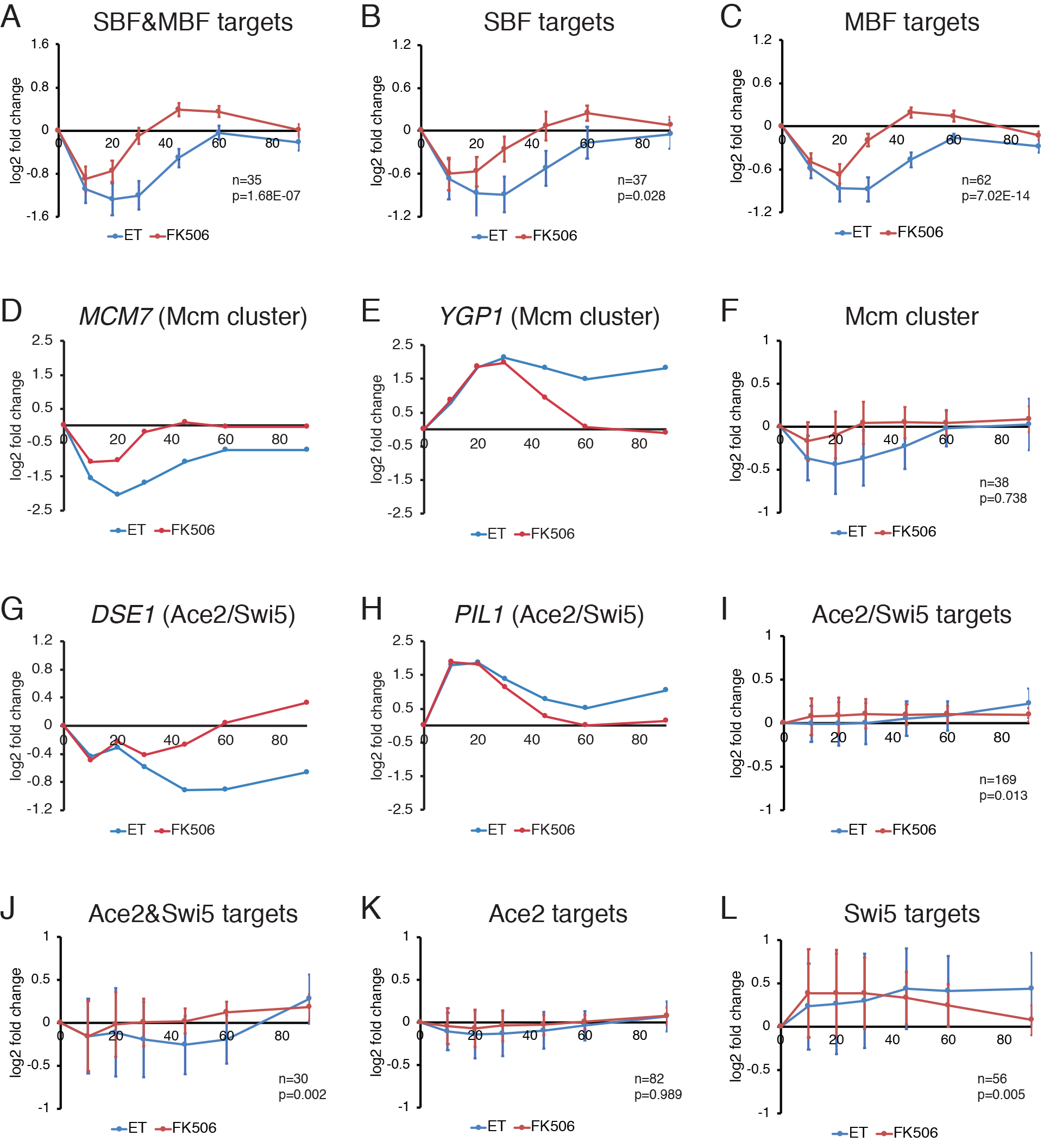

Supplement: S3 Fig — (A-C) Average expression of SBF/MBF target genes from Fig 2A and 2E, divided into subsets of genes regulated by both SBF and MBF (A), only SBF (B), or only MBF (C). (D) Expression of the Mcm cluster gene MCM7 from RNA-seq experiments. (E) Expression of Mcm cluster gene YGP1 from RNA-seq experiments. Note that YGP1 is also a Hog1-regulated gene. (F) Average expression of all Mcm cluster genes shown in Fig 2A. Error bars indicate the 95% confidence interval. Number of genes (n) and the adjusted p-value indicating the significance of the difference between ET and FK506 curves are included. (G) Expression of Ace2/Swi5 target gene DSE1 from RNA-seq experiments. (H) Expression of Ace2/Swi5 target gene PIL1 from RNA-seq experiments. Note that PIL1 is also a Hog1-regulated gene. (I) Average expression of all Ace2/Swi5 target genes shown in Fig 2A. Error bars indicate the 95% confidence interval. Number of genes (n) and the adjusted p-value indicating the significance of the difference between ET and FK506 curves are included. (J-L) Average expression of Ace2/Swi5 target genes from Fig 2A and (I), divided into subsets of genes regulated by both Ace2 and Swi5 (J), only Ace2 (K), or only Swi5 (L). For all parts, lists of genes and values are included in S1 Data. (TIF) [file pgen.1008600.s003.tif]

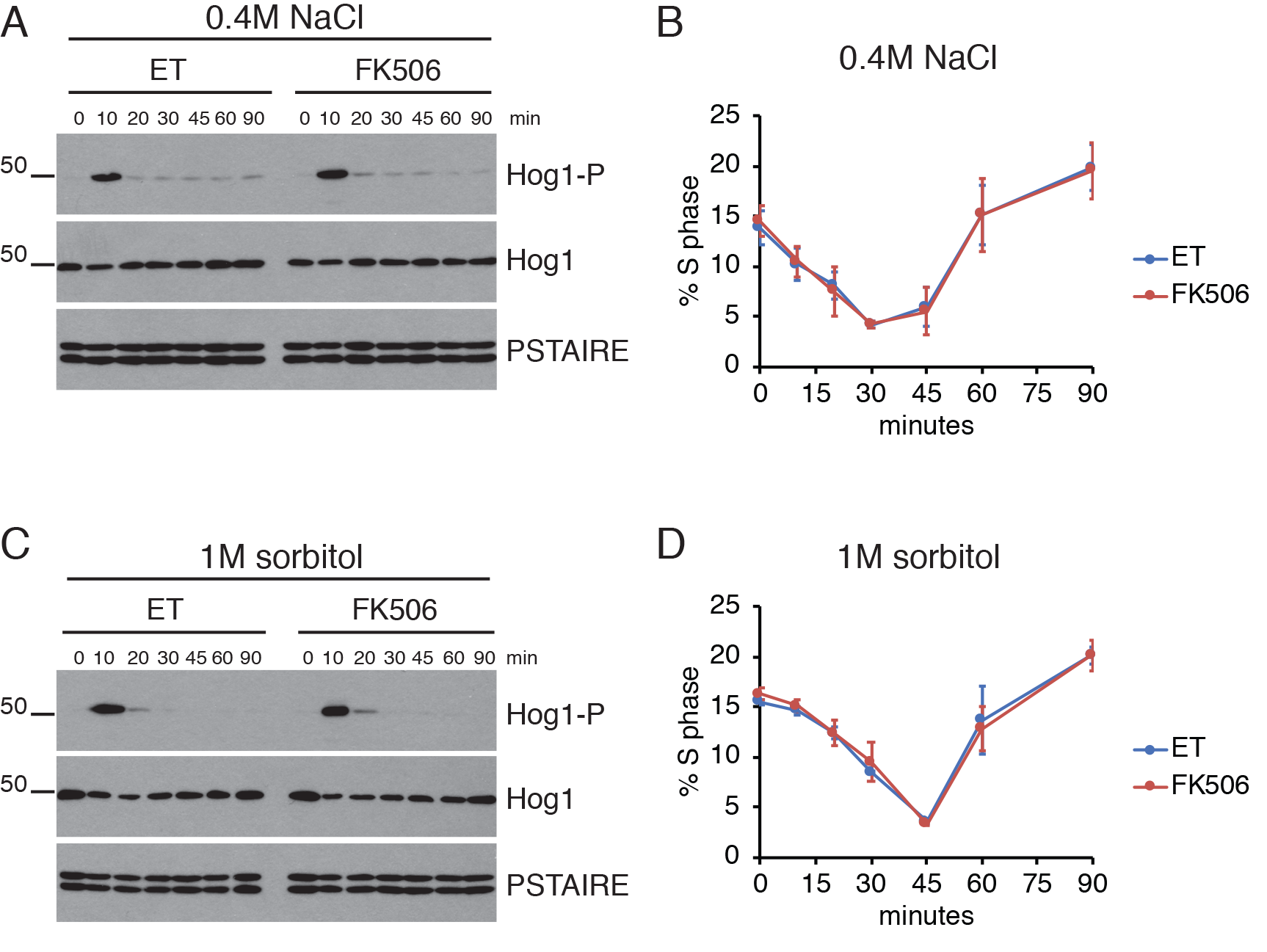

Supplement: S4 Fig — crz1Δ cells were pre-treated with ET buffer or FK506 for 15 minutes before the addition of 0.4M NaCl (A-B) or 1M sorbitol (C-D). Phosphorylated Hog1 (Hog1-P), total Hog1 and PSTAIRE (loading control) were monitored by Western blot (A, C) and percentage of cells in S-phase were quantified (B-D). For parts B & D, an average of n = 3 experiments are shown and error bars indicate standard deviations. Cell cycle positions were measured using a Guava EasyCyte flow cytometer. (TIF) [file pgen.1008600.s004.tif]

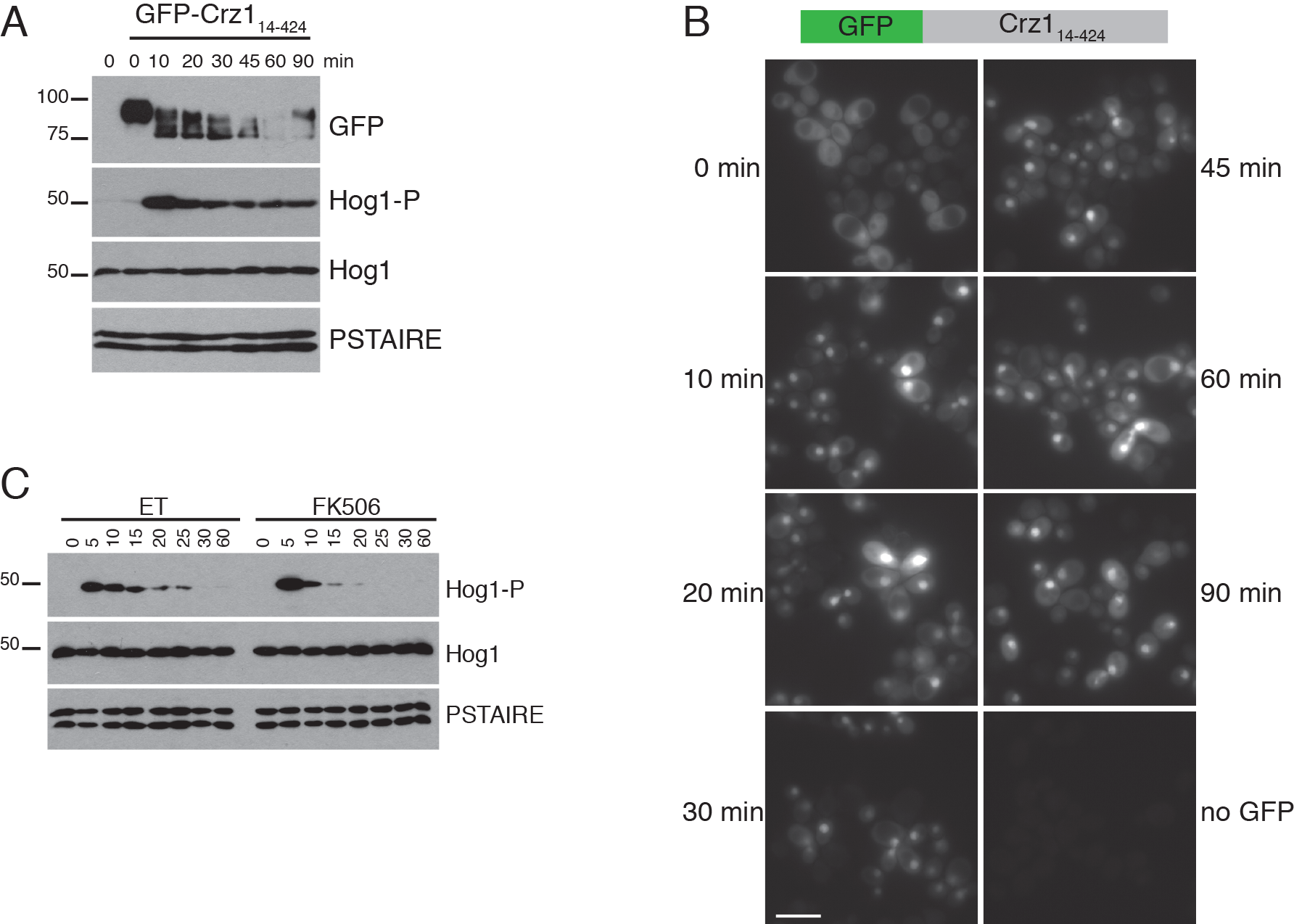

Supplement: S5 Fig — (A) crz1Δ cells expressing GFP fused to a portion of Crz1 that lacks the DNA binding domain (residues 14–424) were treated with CaCl2 for the indicated number of minutes. Dephosphorylation of the GFP-fusion protein was monitored by Western blot and confirms that CN is active throughout the 90-minute time course and correlates with the maintenance of Hog1 phosphorylation (Hog1-P). (B) Cells from (A) were imaged at the indicated time points to confirm that the GFP-Crz1 reporter is nuclear in most cells throughout the 90-minute time course. Cells without the GFP reporter are shown as a negative control. Scale bar represents 10μm. (C) Hog1 activation in wild-type cells is regulated by CN. Wild-type (CRZ1) cells were arrested for 3 hours in G1 with alpha factor, then pre-treated with ET buffer or FK506 for 15 minutes before releasing from the arrest into medium containing 200mM CaCl2. Samples were collected after the indicated number of minutes and Hog1 activation monitored by Western blot (Hog1-P). Total Hog1 and PSTAIRE (loading control) are shown. (TIF) [file pgen.1008600.s005.tif]

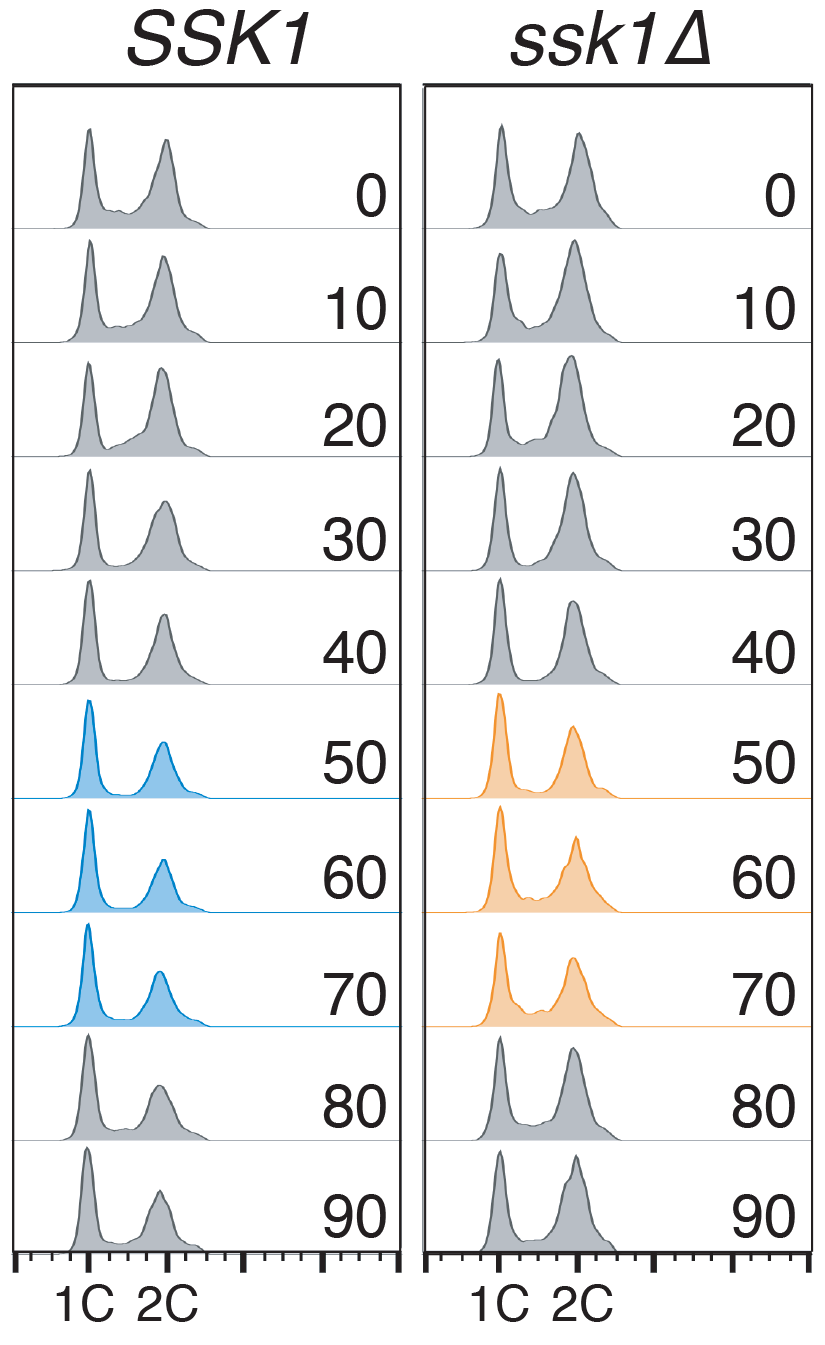

Supplement: S6 Fig — FACS plots from representative CaCl2 time course in crz1Δ SSK1 and crz1Δ ssk1Δ cells shown in Fig 4D. (TIF) [file pgen.1008600.s006.tif]

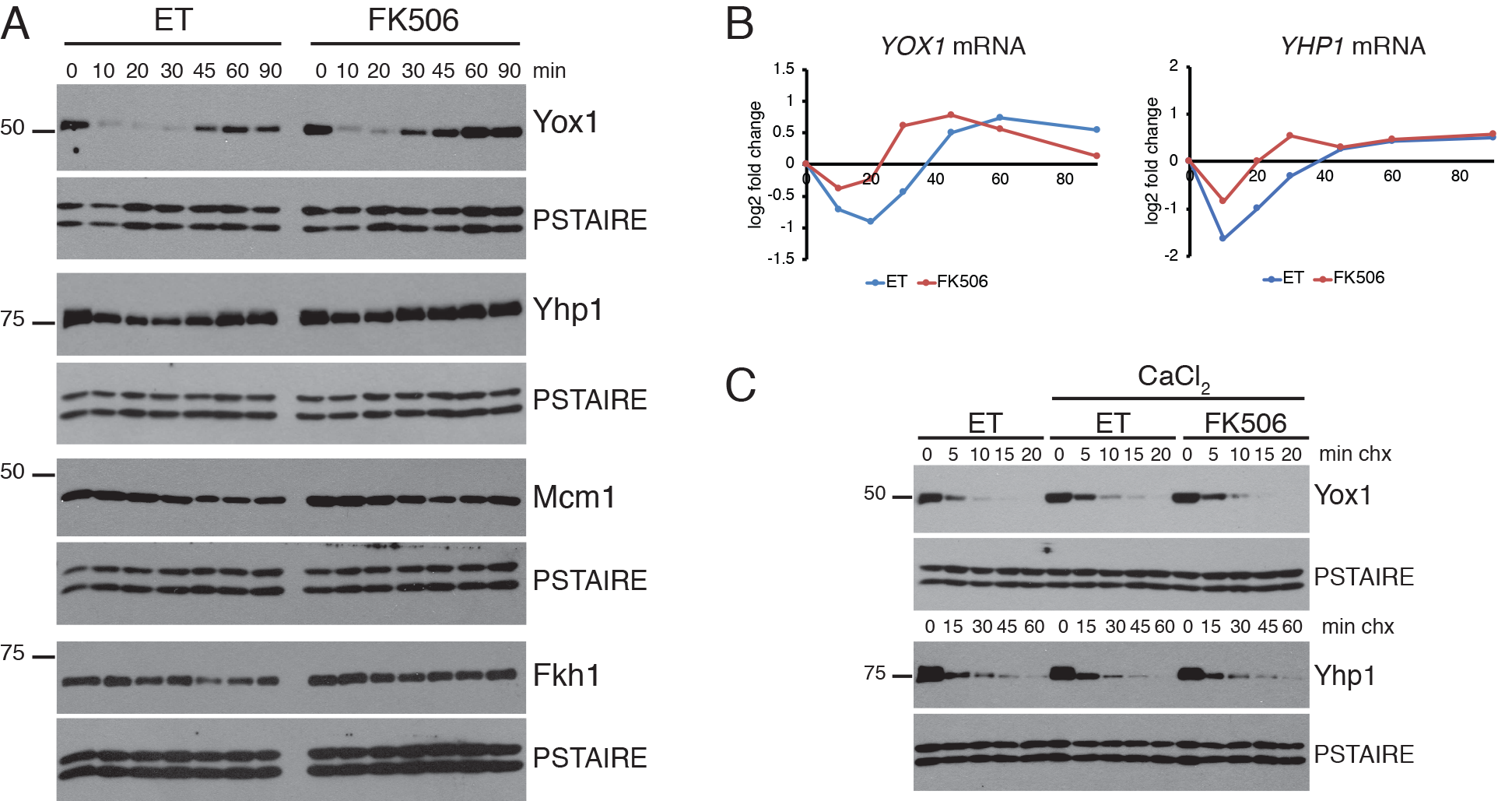

Supplement: S7 Fig — (A) Strains expressing the indicated tagged TFs were pretreated with ET buffer or FK506 for 15 minutes before the addition of CaCl2. Samples were collected for Western blotting at the indicated time points. Western blots were performed for a 3V5 tag on Fkh1, Mcm1, and Yox1 or a 13MYC tag on Yhp1. For all experiments PSTAIRE blots are shown as a loading control. (B) Expression of TF mRNAs in response to CaCl2. Shown are log2 fold change values, compared to the 0-minute time point, from RNA-seq experiments described in Fig 2. (C) Cycloheximide-chase assays of the indicated TF proteins. Cells expressing tagged TF proteins from (A) were pretreated with ET buffer or FK506 for 10 minutes, CaCl2 was added for an additional 5 minutes, then cycloheximide was added (0 minutes) and samples collected at the indicated time points for Western blot. (TIF) [file pgen.1008600.s007.tif]

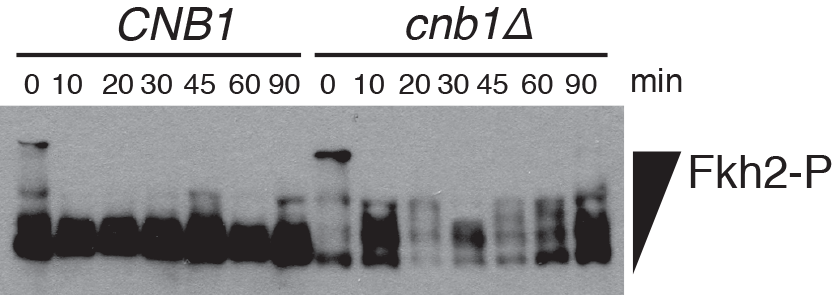

Supplement: S8 Fig — crz1Δ CNB1 and crz1Δ cnb1Δ strains were pretreated with ET buffer or FK506 for 15 minutes before the addition of CaCl2. Samples were collected at the indicated time points and Phos-tag Western blot performed for a 3FLAG tag on Fkh2. (TIF) [file pgen.1008600.s008.tif]
