# Supplementary material for: The coordinate actions of calcineurin and Hog1 mediate the stress response through multiple nodes of the cell cycle network
Source: PLoS Genet. 2020 Apr 28;16(4):e1008600. doi: 10.1371/journal.pgen.1008600 (PMC7209309; doi:10.1371/journal.pgen.1008600)
Supplement: S1 Table — (PDF) [file pgen.1008600.s009.pdf]

S1 Table. CN-regulated genes after 10 minutes $CaCl_2$ treatment

gene	ET/FK506 t=10*	BH adj p-value	gene cluster
BAG7	-1.856960889	0.000967319	
HAP4	-1.163055122	8.23E-07	
REG2	-1.150626613	0.042108723	Ace2/Swi5
NRM1	-1.141897458	0.007858729	SBF/MBF
ZEO1	-1.0536199	0.029144541	
CLB2	-1.031467576	0.000206166	Fkh2/Ndd1
SUR7	-0.949017459	0.000268532	
POG1	-0.914706391	0.041214538	
CDC5	-0.888550191	0.00199479	Fkh2/Ndd1
FRK1	-0.843561961	0.000975715	Fkh2/Ndd1
COS111	-0.834665457	0.012939887	
ALK1	-0.796044016	0.00199479	Fkh2/Ndd1
YNL058C	-0.765573748	0.037184485	Fkh2/Ndd1
SWI5	-0.749489208	0.00199479	Fkh2/Ndd1
TPO3	-0.721860491	0.042108723	Fkh2/Ndd1
MIG1	-0.703374214	0.029144541	
IRC8	-0.682302946	0.009439705	Fkh2/Ndd1
PMA1	-0.648080526	0.023655503	
SRC1	-0.638086785	0.00199479	Fkh2/Ndd1
ASE1	-0.617232544	0.0027527	Hcm1
YDR133C	-0.6099885	0.048012708	
FKH2	-0.609401994	0.029144541	Hcm1
ACE2	-0.605854216	0.0333304	Fkh2/Ndd1
MRH1	-0.56727327	0.042108723	MCM cluster
PHO90	-0.553456027	0.019177709	
ECM33	-0.469376696	0.029144541	Hcm1
TSC10	-0.421085289	0.041068935	
HST3	-0.339988525	0.016264338	Fkh2/Ndd1
MET22	0.481421259	0.028290041	
YAP5	0.651389782	0.04214145	
MET16	0.911730288	0.004058974	
ATG41	1.132156074	0.009224367	
STR3	1.572852266	0.00199479	

*Shown are log2 fold change values of ET-10min/FK506-10min
