# Supplementary material for: The coordinate actions of calcineurin and Hog1 mediate the stress response through multiple nodes of the cell cycle network
Source: PLoS Genet. 2020 Apr 28;16(4):e1008600. doi: 10.1371/journal.pgen.1008600 (PMC7209309; doi:10.1371/journal.pgen.1008600)
Supplement: S2 Table — (PDF) [file pgen.1008600.s010.pdf]

S2 Table. Strain table

strain	genotype
YHA146	*MATa ura3Δ0 leu2Δ0 his3Δ0 met15Δ0 crz1Δ::KanMx HCM1-3HA-HIS3*
YHA147	*MATa ura3Δ0 leu2Δ0 his3Δ0 met15Δ0 crz1Δ::KanMx hcm1-ΔPSIEIQ-3HA-HIS3*
YCL34	*MATa his3Δ ura3Δ leu2Δ met15Δ lys2Δ crz1Δ::LEU2 MCM1-3V5-KanMX*
YMF1	*MATa his3Δ0 ura3Δ0 leu2Δ0 lys2Δ0 met15Δ0 crz1Δ::LEU2 cnb1Δ::KanMX HCM1-3HA-HIS3*
YMF7	*MATa his3Δ0 ura3Δ0 leu2Δ0 crz1Δ::LEU2*
YCL37	*MATa his3Δ ura3Δ leu2Δ met15Δ crz1Δ::LEU2 NDD1-3V5-KanMX*
YCL153	*MATa ura3Δ0 leu2Δ0 his3Δ0 met15Δ0 crz1Δ::LEU2 sho1Δ::URA3 NDD1-3V5-KanMx*
YCL154	*MATa ura3Δ0 leu2Δ0 his3Δ0 met15Δ0 crz1Δ::LEU2 ssk1Δ::URA3 NDD1-3V5-KanMx*
YJB622	*MATa ura3Δ0 leu2Δ0 his3Δ0 met15Δ0 crz1Δ::KanMx HCM1-3HA-HIS3 hog1Δ::LEU2*
YCL99	*MATa his3Δ ura3Δ leu2Δ met15Δ crz1Δ::LEU2 FKH2-3FLAG-Hyg*
YMF42	*MATa his3Δ ura3Δ leu2Δ met15Δ crz1Δ::LEU2 swe1Δ::URA3 NDD1-3V5-KanMX*
YMF44	*MATa his3Δ ura3Δ leu2Δ met15Δ crz1Δ::LEU2 hog1Δ::HIS3 NDD1-3V5-KanMX*
YMF45	*MATa his3Δ ura3Δ leu2Δ met15Δ crz1Δ::LEU2 hog1Δ::HIS3 swe1Δ::URA3 NDD1-3V5-KanMX*
YMF41	*MATa his3Δ ura3Δ leu2Δ met15Δ FKH2-3FLAG-Hyg crz1Δ::LEU2 swe1Δ::URA3*
YMF46	*MATa his3Δ ura3Δ leu2Δ met15Δ crz1Δ::LEU2 hog1Δ::HIS3 FKH2-3LAG-Hyg*
YMF47	*MATa his3Δ ura3Δ leu2Δ met15Δ crz1Δ::LEU2 hog1Δ::HIS3 swe1Δ::URA3 FKH2-3FLAG-Hyg*
YBL176	*MATa ura3Δ0 leu2Δ0 his3Δ0 met15Δ0 HCM1-3HA-HIS3*
YMF55	*MATa ura3Δ0 leu2Δ0 his3Δ0 lys2Δ0 crz1Δ::LEU2 +pAMS478*
YMF57	*MATa ura3Δ0 leu2Δ0 his3Δ0 lys2Δ0 crz1Δ::LEU2 +pRS316*
MW836a	*MATa ura3Δ0 leu2Δ0 his3Δ0 met15Δ0*
YCL31	*MATa his3Δ ura3Δ leu2Δ met15Δ crz1Δ::LEU2 FKH1-3V5-KanMX*
YCL43	*MATa his3Δ ura3Δ leu2Δ met15Δ crz1Δ::LEU2 YOX1-3V5-KanMX*
YCL53	*MATa his3Δ ura3Δ leu2Δ met15Δ crz1Δ::LEU2 YHP1-13MYC-KanMx*
YMF92	*MATa his3Δ ura3Δ leu2Δ lys2Δ crz1Δ::LEU2 cnb1Δ::KanMx Fkh2-3FLAG-HYG*

All strains are in the BY4741 background.
