# Supplementary material for: The coordinate actions of calcineurin and Hog1 mediate the stress response through multiple nodes of the cell cycle network
Source: PLoS Genet. 2020 Apr 28;16(4):e1008600. doi: 10.1371/journal.pgen.1008600 (PMC7209309; doi:10.1371/journal.pgen.1008600)
Supplement: S3 Table — (PDF) [file pgen.1008600.s011.pdf]

## S3 Table. Primer table

| gene | primer name | sequence |
| --- | --- | --- |
| *ACT1* | ACT1fwd | ATGAAGTGTGATGTCGATGTCC |
| | ACT1rev | CCAATCCAGACGGAGTACTTTC |
| *STL1* | STL1fwd | TTGCGGTATTTCATCACTATCG |
| | STL1rev | CACTACAGTTGCGTGTCTGTCA |
| *YOX1* | YOX1fwd | ATTTGCTTTCATCACACACTCG |
| | YOX1rev | AACTCAATTCGTTTCTCCTTCG |
| *CLN1* | CLN1fwd | CTTTGGTTAGCGGCCAAAAC |
| | CLN1rev | AGAAAGGCGTGGAATACGAG |
| *CLB2* | CLB2fwd | TGCATGTACGGAAGATGAAATC |
| | CLB2rev | AAGAATTTGGCAAGAGTTCGAG |
| *CDC5* | CDC5fwd | ATGTCCCATCCAAATATCGTTC |
| | CDC5rev | AATTCCATTAATGAACCGTTGG |
| *CDC20* | CDC20fwd | GCGGTAACCGTTCTGTACTTTC |
| | CDC20rev | GGGACGTTTGGAGTTTCTAATG |
